# Elucidating gastric cancer mechanisms and therapeutic potential of Adociaquinone A targeting EGFR: A genomic analysis and Computer Aided Drug Design (CADD) approach

**DOI:** 10.1111/jcmm.70133

**Published:** 2024-10-21

**Authors:** Mariam Abdulaziz Alkhateeb, Nada H. Aljarba, Qudsia Yousafi, Fatima Anwar, Partha Biswas

**Affiliations:** ^1^ Department of Biology, College of Science Princess Nourah bint Abdulrahman University Riyadh Saudi Arabia; ^2^ Department of Biosciences COMSATS University Islamabad, Sahiwal Campus Sahiwal Pakistan; ^3^ Laboratory of Pharmaceutical Biotechnology and Bioinformatics, Department of Genetic Engineering and Biotechnology Jashore University of Science and Technology Jashore Bangladesh

**Keywords:** CMNPD, hub genes, miRNA, molecular docking, survival analysis

## Abstract

Gastric cancer predominantly adenocarcinoma, accounts for over 85% of gastric cancer diagnoses. Current therapeutic options are limited, necessitating the discovery of novel drug targets and effective treatments. The Affymetrix gene expression microarray dataset (GSE64951) was retrieved from NCBI‐GEO data normalization and DEGs identification was done by using R‐Bioconductor package. Gene Ontology (GO) analysis of DEGs was performed using DAVID. The protein–protein interaction network was constructed by STRING database plugin in Cytoscape. Subclusters/modules of important interacting genes in main network were extracted by using MCODE. The hub genes from in the network were identified by using Cytohubba. The miRNet tool built a hub gene/mRNA‐miRNA network and Kaplan–Meier‐Plotter conducted survival analysis. AutoDock Vina and GROMACS MD simulations were used for docking and stability analysis of marine compounds against the 5CNN protein. Total 734 DEGs (507 up‐regulated and 228 down‐regulated) were identified. Differentially expressed genes (DEGs) were enriched in processes like cell–cell adhesion and ATP binding. Eight hub genes (EGFR, HSPA90AA1, MAPK1, HSPA4, PPP2CA, CDKN2A, CDC20, and ATM) were selected for further analysis. A total of 23 miRNAs associated with hub genes were identified, with 12 of them targeting PPP2CA. EGFR displayed the highest expression and hazard rate in survival analyses. The kinase domain of EGFR (PDBID: 5CNN) was chosen as the drug target. Adociaquinone A from *Petrosia alfiani*, docked with 5CNN, showed the lowest binding energy with stable interactions across a 50 ns MD simulation, highlighting its potential as a lead molecule against EGFR. This study has identified crucial DEGs and hub genes in gastric cancer, proposing novel therapeutic targets. Specifically, Adociaquinone A demonstrates promising potential as a bioactive drug against EGFR in gastric cancer, warranting further investigation. The predicted miRNA against the hub gene/proteins can also be used as potential therapeutic targets.

## 1 INTRODUCTION

Gastric cancer has shown a worldwide decline since the 1970s which is attributed to effective *Helicobacter pylori* eradication strategies and^1^ and advances in refrigeration technology.^2^, ^3^, ^4^ Despite these improvements, gastric cancer continues to pose a significant global health challenge, particularly in East Asia, where it accounted for over 1 million cases in 2020.^5^, ^6^ Adenocarcinoma, the most common and dangerous type of gastric cancer, accounts for more than 85% percent of the gastric cancer diagnosis. Its prevalence has been significantly reduced in developed nations through targeted prevention strategies.^7^, ^8^ However, it remains a critical health concern in less developed regions.^9^, ^10^, ^11^ The cumulative risk of developing gastric cancer from birth to age of 74 years is estimated more than 1.87%. The disease disproportionately affects males, who represent 68% of all cases, compared to 32% in females.^12^ According to the Human Development Index (HDI), the average incidence rate of gastric cancer is 20 per million in men and 6.6 per million in women.^13^ It is the third most common reason for cancer mortality globally, owing 8.8% of all cancer‐related deaths, in both sexes.^14^, ^15^, ^16^

**FIGURE 1:**
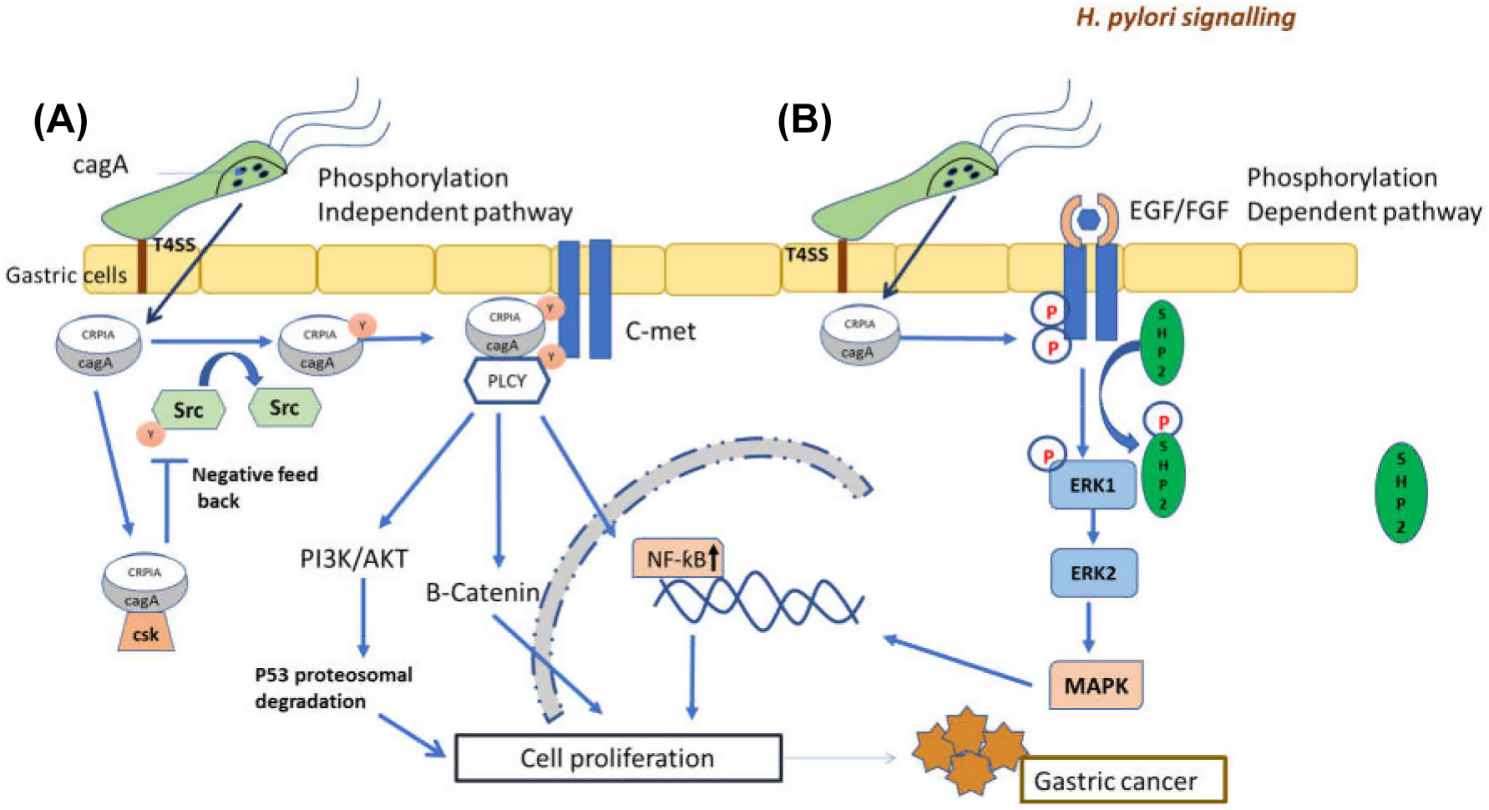
Phosphorylation dependent and independent pathway through CagA. CagA cytotoxin associated gene follows two pathways for causing cellular proliferation which is one is (A) phosphorylation independent pathway in which it triggers AKT pathway (also known as protein kinase B or PKB) leading to p53 proteasomal degradation, which leads to cellular proliferation and ultimately to gastric cancer. (B) Phosphorylation dependent pathway which follows the MAP (mitogen‐activated protein) kinase pathway which also leads to cellular proliferation and ultimately to gastric cancer (*Source*: Sonkar et al., 2022.^23^

Age‐related accumulation of somatic mutations can potentially increase the frequency of gastric cancer occurrence.^17^ Obesity^18^ and tobacco consumption are important causes of development of this cancer in males.^19^ Extensive alcohol consumption results in high risk of gastric cancer development both in smokers and for non‐smokers.^19^, ^20^, ^21^ Patients with *H. pylori* infection have been found to have seven times higher risk compared to non‐infected persons.^12^ The most prevalent source of gastric cancer is *H. pylori* CagA‐positive strains. The CagA protein (encoded by CogA gene) are secreted by type 4 secretion system (T4SS) and penetrate to gastric epithelial cells.^22^ It undergoes tyrosine phosphorylation by Protooncogene tyrosine‐protein kinase (Src), at EPIYA motif. This phosphorylated CagA protein interacts with multiple signalling host protein and serves a function of scaffold. This flow of signals result in differentiation and proliferation of gastric epithelial cells. Another infection pathway is phosphorylation independent in which activation occurs without phosphorylation of tyrosine residues. CagA also has Conserved repeat responsible for phosphorylation‐independent activity. The CCRPIA motif present on CagA interacts with hepatocyte growth factor, scatter factor receptor c‐Met. This interaction results indefinite cell proliferation in gastric cancer^23^ (Figure 1). Whether or not abolition of *H. pylori* in the lack of dysplastic or neoplastic tissues stops development of gastric cancer is unresolved.^24^ It is currently the fourth most common cause of cancer mortality worldwide but prediction for the treatment of this disease is still poor.^25^ The trends of occurrence are observed sporadic and familial in most of the cases^26^ although hereditary acquisition of this disease is very rare that is 1%–3%.^27^

The overall higher survival rate was observed in cases where combination of treatment techniques were used compared of those treated with single technique. Hu et al.,^28^ in a study found that hazard ratio (HR), for the patients in supportive care only>surgery alone>surgery plus chemotherapy. This shows the two treatments, surgery + chemotherapy, can be proved effective approach for treating this disease. An appropriate, least toxic and nonpromiscuous drug combined with other treatments can be value addition to cure this disease. The reduction in the intake of chemically preserved products also helps to reduce the risk of for this disease.^29^

Numerous molecular biomarkers, identified through high‐throughput screening techniques, have emerged as effective therapeutic targets. However, the underlying genetic mechanisms remain largely unexplored. Traditional approaches, focusing on single genes or factors, can clarify specific aspects of carcinoma progression but fail to consider the synergistic effects of multiple genes and pathways. This oversight is crucial in understanding cancer's onset, progression and treatment. Significantly, integrating multiple datasets and analytical methods could enhance the accuracy and scientific validity of research findings. Such integration would provide a more comprehensive understanding of the differentially expressed genes (DEGs) involved in cancer, leading to more informed subsequent research.^30^

Currently, treatment for gastric cancer primarily involves surgery and conventional chemotherapy, yet the prognosis remains poor. A deeper comprehension of the genetic mechanisms underlying gastric cancer is essential. This understanding could lead to the identification of critical biomarkers and the development of novel, safer drug therapies aimed at reducing the incidence of this disease. In related research, WDR12 has been identified as a potential biomarker across various human cancers, highlighting its significance in cancer research.^31^

We utilized bioinformatics methods to predict biomarkers for gastric cancer by analysing publicly available microarray expression data (GSE64951) from the Gene Expression Omnibus (GEO) database. The differentially expressed genes (DEGs) were subjected to Gene Ontology enrichment analysis, using the DAVID tool, to understand their biological implications.

We further explored these DEGs by constructing a protein–protein interaction (PPI) network to identify crucial hub genes. Additionally, a miRNA network was developed for these hub genes/mRNA, and survival analyses were conducted to determine their significant impact on the pathogenesis and prognosis of gastric cancer.

One notable finding is the significantly elevated expression levels of epidermal growth factor receptor (EGFR) proteins in gastric cancer tumour cells, ranging between 40% and 60%. EGFR has been identified as a critical therapeutic target and its domain structure was specifically targeted in our drug design studies against gastric cancer.^32^

EGFR inhibition therapy, with drugs like erlotinib and gefitinib, has become a standard cancer treatment approach.^33^ However, these inhibitors are often associated with dermatological side effects.^34^ To address these concerns, we have identified a novel, non‐toxic EGFR inhibitor derived from marine life sources. This promising drug candidate molecule offers a safer alternative for treating gastric cancer, potentially minimizing the side effects commonly seen with current therapies.

## 2 MATERIALS AND METHODS

### 2.1 Differentially expressed genes analysis

#### 2.1.1 Collection normalization and statistical analysis of dataset for identification of differentially expressed genes (DEGs)

The Affymetrix gene expression microarray dataset (GSE64951) was downloaded from Gene Expression Omnibus (GEO: www.ncbi.nlm.nih.gov/geo/) and normalized by R Bioconductor package. The normalized dataset was statistically analysed by using MS Excel in order to get the *p* values, adjusted *p* values, Log_2_ Fold change (Log_2_FC). The *p* value is the probability which measures how likely it is that any observed difference between groups is by chance.^35^ The probability of finding a significant *p* value by chance is increased with larger datasets which results in false positive results. The False Discovery Rate (FDR) adjusts the *p* values according to the number of total tests and adjusted *p* values are obtained.^36^ Fold change (FC) is often used to select the DEGs in a microarray dataset with two biological conditions (test/diseased and control/healthy).^37^ It is calculated as a ratio of averages from control and test sample.^38^ However, log‐ratios are often used for analysis and visualization of fold changes. The logarithm to base 2 is most commonly used, as it is easy to interpret, for example a doubling in the original scaling is equal to a log_2_ fold change of 1.^39^ The thresholds were set as, adjusted *p* <0.05 and |log_2_ fold change (log_2_FC)| ≥1 for identification of DEGs.^40^ Positive value of log_2_ FC represent upregulated gene while negative value is assigned to downregulated genes.^41^ The upregulated and downregulated genes are called differentially expressed genes (DEGs).^41^

#### 2.1.2 Gene ontology enrichment analysis of DEGs

Functional annotation of the DEGs was performed using the GO terms that helps filter out their roles in different biological processes (BP). The list of ENTERZ gene IDs was uploaded to Database for annotation, visualization and integrated discovery (DAVID)^42^ to perform the functional annotation highlighting the biological importance behind the large set of genes.^43^ Three categories of Gene ontology (GO) that is, BP, molecular functions (MF) and cellular components (CC) were selected for analysing the DEGs. The DEGs involved in each category were selected at a threshold of *p* <0.005.

#### 2.1.3 Protein–protein interaction network, modules construction and pathway enrichment analysis of DEGs

Protein–protein interaction (PPI) network of DEGs involved in gastric cancer was created by STRING database plugin in Cytoscape 3.8.2 (https://cytoscape.org/). The table with Log_2_FC value was created by assigning 0 to negative (downregulated DEGs) and 1for positive values (upregulated DEGs) and imported in Cytoscape. The upregulated DEGs (nodes) assigned red and down regulated as green. The functionally important sub‐clusters (modules) in the main network were extracted by Molecular Complex Detection (MCODE) application in Cytoscape. The modules generated were analysed by saving the output in the .doc form and MCODE network. The top three modules were selected for pathways enrichment analysis. The list of DEGs from selected three modules was imported to the ClueGO plugin of Cytoscape to generate and envision a functionally grouped network of terms/pathways. On the basis of the similarity among the related genes, ClueGO demonstrates the selected terms in an annotated network that is functionally grouped. The node size of the terms shows the statistical significance. Further kappa statistics was used in order to calculate the degree of correspondence between edges or terms. The calculated value of the kappa score that is, ≥ 0.3 shows the essential functional group.^44^

#### 2.1.4 Identification of hub genes and their association with other genes in functional network

CytoHubba, Cytoscape plugin, was used for identification of the hub genes in the PPI network. CytoHubba analysed the network on the basis of 12 statistical methods that is, MCC, DMNC, MNC, Degree, EPC, BottleNeck, Eccentricity, Closeness, Radiality, Betweenness, Stress, and Clustering Coefficient, for identification of hub genes. The consensus DEGs identified by more than 50% of the methods were selected as hub genes. The list of Hub genes was imported to another Cytoscape plug in GeneMANIA to find their association with other functionally important human genes.

#### 2.1.5 miRNA network construction for hub genes

MicroRNAs (miRNA) are a key modulator in tumour suppression and mRNA degradation in cellular pathway for controlling expression of target genes. They have the ability to target 10 to 100 genes at a time. Therefore, miRNAs are important as predictive biomarkers and therapeutic targets for cancer therapeutics. The list of hub genes was submitted to mirNet (https://www.mirnet.ca/) and miRNA for *Homo sapiens* specific for gastric cancer were selected. The mRNA/hub gene‐ miRNA network was generated showing miRNA nodes directing to target mRNA. The network file ‘mirnet.graphml’ was downloaded and imported into Cytoscape for network construction and visualization.

#### 2.1.6 Survival analysis of hub genes

The survival analysis for essential genes involved in breast, lung, ovarian, liver and gastric cancer is available on Kaplan–Meier‐Plotter database (https://kmplot.com/analysis/). The predictive value of hub genes in gastric cancer patients was assessed using the Kaplan–Meier‐Plotter. The log‐rank *p* value and the HR with 95% confidence interval (CI) were calculated for plotting The Kaplan–Meier survival curves. The GEPIA database was used for additional analysis. The findings were displayed in boxplots in order to validate the mRNA (hub genes) expression levels in cancer and normal gastric tissues.

### 2.2 Computer aided drug design (CADD)

#### 2.2.1 Retrieval of receptor protein

The amino acid FASTA sequence file for EGFR was downloaded from UniProt. The FASTA sequence of protein was submitted to Interpro‐EMBL‐EBI (https://www.ebi.ac.uk/interpro/), server for scanning its active domain. The FASTA sequence of the selected domain was submitted to NCBI BLASTp server for searching the closest template for 3D structure prediction.

**FIGURE 2:**
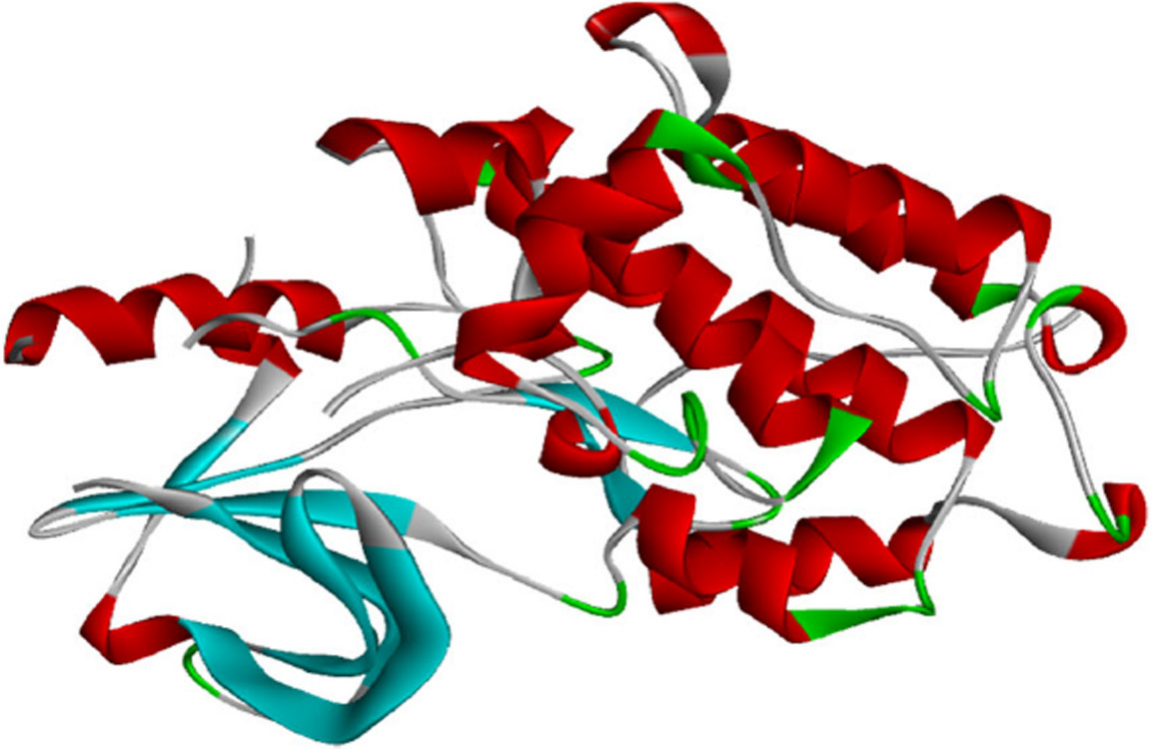
Three‐dimensional structure of chain A of EGFR kinase domain (PDB ID: 5CNN).

EGFR kinase domain mutant I682Q (PDBID: 5CNN) showed 100% query coverage and percent identity with the domain sequence. The 3D structure of 5CNN was downloaded from Protein Data Bank (PDB: https://www.rcsb.org/). The extra chains, water molecules and the non‐standard amino acids were removed by using UCSF Chimera^45^ (Figure 2).

#### 2.2.2 Ligand compound structures

The 3D and 2D structure of the marine life originated chemical compounds, to be used as inhibitors of EGFR were downloaded from the Comprehensive Marine Natural Products Database (CMNPD)^46^ (Table S1). The toxicity analysis of compounds was done by using AdmetSAR (http://lmmd.ecust.edu.cn/admetsar2) and Datawarior (https://openmolecules.org/datawarrior/) while drug‐likeness was determined by the drug likeness tool (DruLiTo).^47^ The nontoxic and drug like compounds following Lipinski's rule of 5 and Veber rules were selected as ligands for ligand‐receptor molecular docking with 5CNN.

#### 2.2.3 Ligand‐receptor molecular docking

The ligand and receptor (5CNN) files were prepared by MGL tools of AutoDock Vina and saved in pdbqt format. Selection of grid was done for blind docking and dimensions were saved in config.doc file. The docking was performed and results were saved as .pdbqt files. The out.pdbqt and receptor.pdb files were imported to PyMOL for the formation of ligand‐receptor docked complex which was saved in pdb format.^48^, ^49^, ^50^ The complex file was visualized in LIGPLOT^51^ for 2D interaction while 3D interactions were visualized by BIOVIA Discovery Studio.^52^ The binding affinities of the complexes were noted from the log files.

#### 2.2.4 Molecular dynamics simulations (MDS)

The top three docked complexes with lowest binding energies and good hydrogen bindings were selected to evaluate stability of interaction for 50 ns time period. The interaction profile for selected ligand‐receptor complexes was studied by running molecular dynamics (MD) simulation using GROMACS 5.0.7 server. Topological and structural evaluations were performed by using force field parameterization PRODRG tool.^53^ The system equilibration for running MD simulation was done by using GROMOS96 43A1 force field.^54^ In order to remove the initial steric conflicts, the systems were first solved using the SPC216 water model in a periodic box (1 nm) and then subjected to energy minimization (steepest descent algorithm for 500 steps). The primary stearic clashes were removed by using 1000 kJ/mol Å2 tolerance. In order to relax the energy‐minimized systems, they were subjected to a 1000 ps equilibration run at constant pressure and temperature. Finally, MD simulations were performed with constant temperature (300 K) and pressure over a time scale of 50 ns (1 atm). All computations were performed using the Particle Mesh Ewald (PME) program to analyse electrostatic interactions. To examine the stability and behaviour of each system, GROMACS modules including g rms, g rmsf, g energy and g h bond functions were used.

## 3 RESULTS

### 3.1 Differentially expressed genes analysis

#### 3.1.1 Collection normalization and statistical analysis of dataset for identification of differentially expressed genes

Total of 734 DEGs retrieved from Affymetrix gene expression microarray dataset (GSE64951) contained 507 upregulated and 228 downregulated genes.

#### 3.1.2 Gene ontology enrichment analysis of DEGs

**FIGURE 3:**
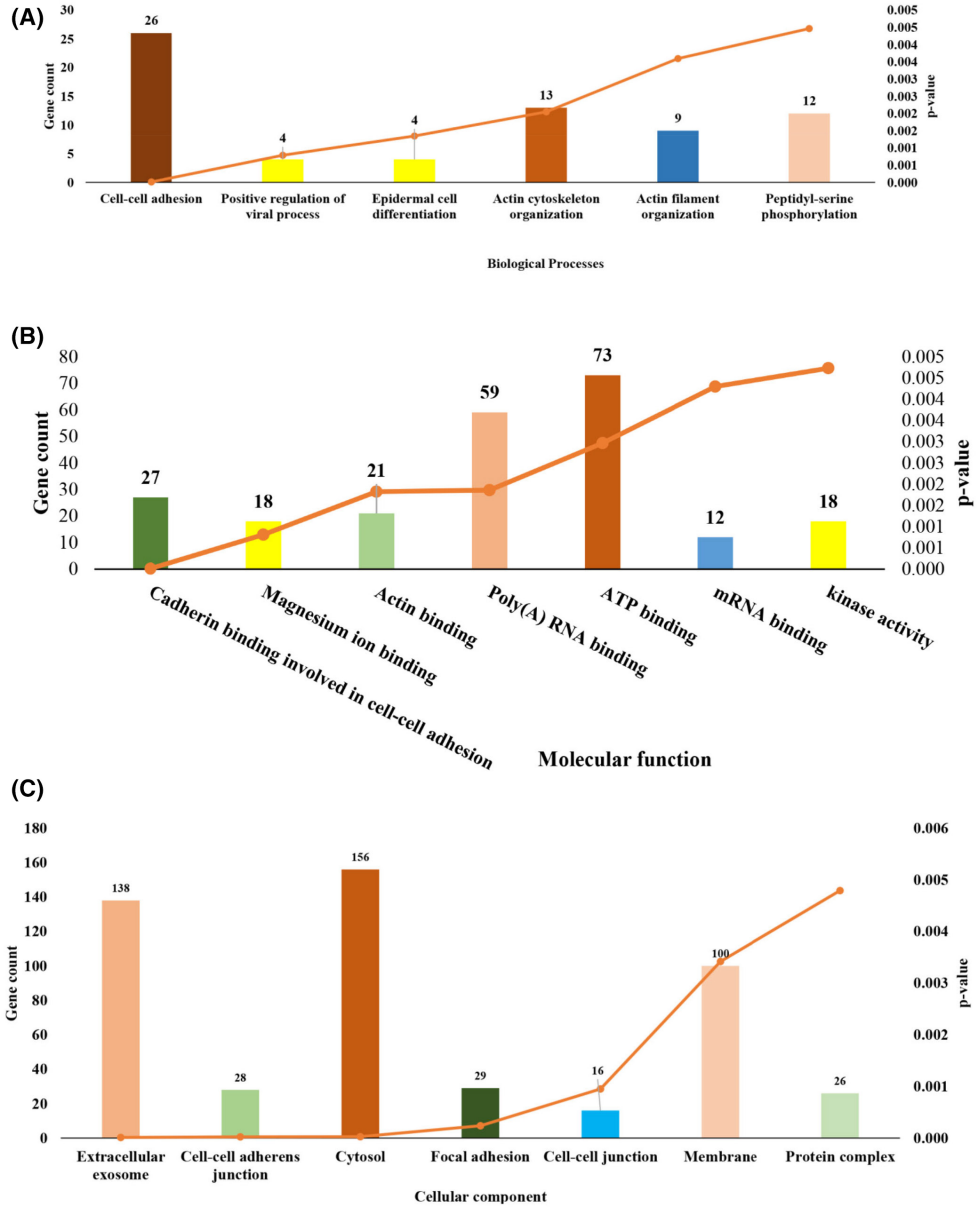
Number of DEGs of gastric cancer (GSE64951) involved in GO categories. (A) Biological processes (B) Molecular Function (C) Cellular components.

Six biological process categories (BP) that is, cell–cell adhesion, positive regulation of viral process, epidermal cell differentiation, actin cytoskeleton organization, actin filament organization and peptidyl‐serine phosphorylation (PSP) were identified (Figure 3A).

The highest number (26) of genes were involved in cell–cell adhesion, followed by 13 and 12 in actin cytoskeleton organization and peptidyl‐serine phosphorylation (PSP) respectively. The predicted MF for DEGs were cadherin binding involved in cell–cell adhesion, magnesium ion binding, actin binding, poly(A) RNA binding, ATP binding, mRNA binding, kinase activity (Figure 3B). The DEGs were found to be localized in seven cell compartments that is extracellular exosome, cell–cell junction, cytosol, focal adhesion, cell–cell junction and membrane protein complex (Figure 3C). The highest numbers (138) of genes were found in extracellular exosome.

#### 3.1.3 Protein–protein interaction network, modules construction and pathway enrichment analysis of DEGs

**FIGURE 4:**
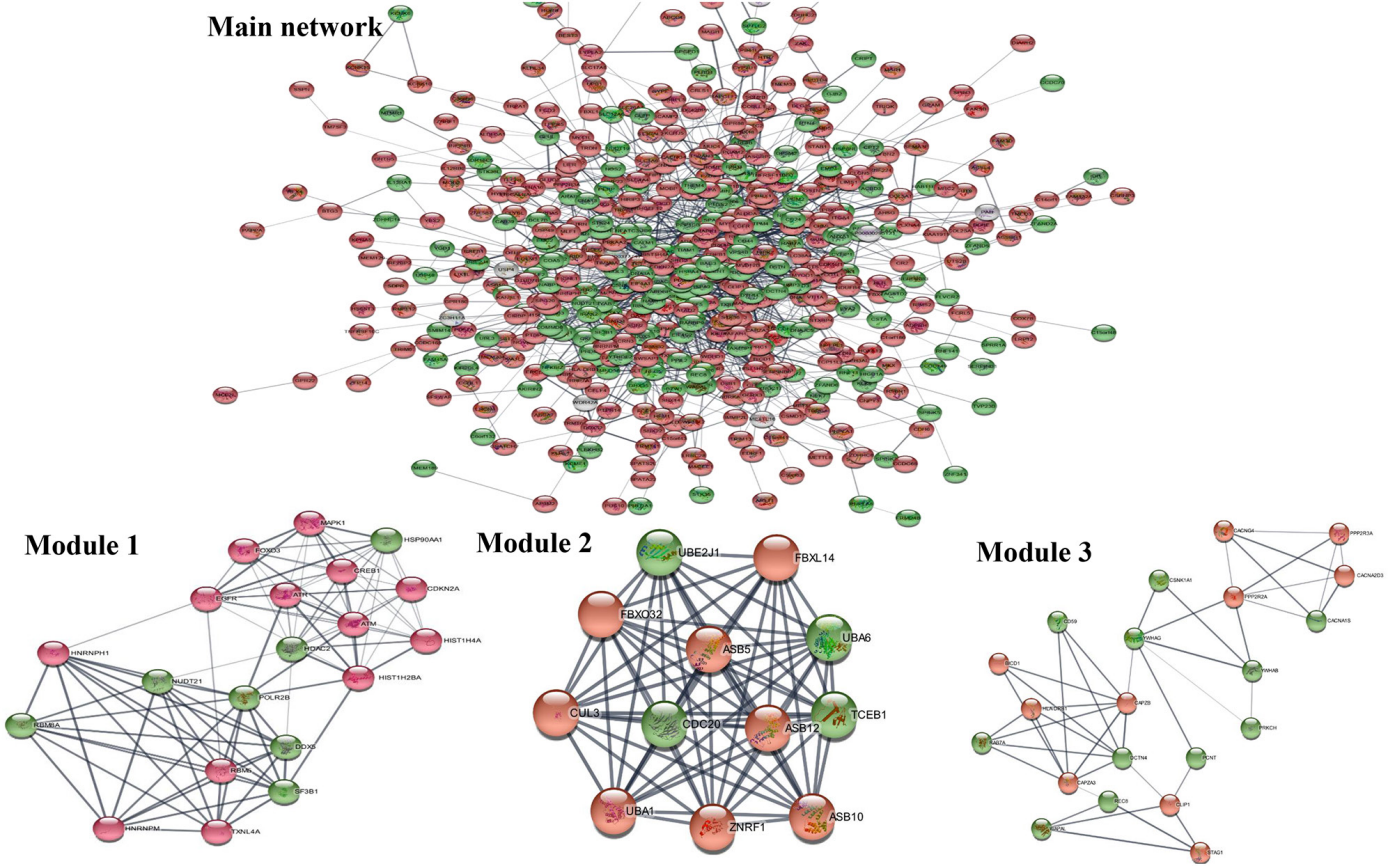
Protein–protein interaction network and network sub‐clusters (modules) for DEGs gastric cancer (GSE64951). Here Green nodes = downregulated genes; Red nodes = upregulated genes.

There were 1658 edges interacting 537 nodes (191 downregulated green and 345 upregulated red nodes) in the network. The sub‐clusters/modules of important interacting genes in the main cluster were predicted by MCODE plugin in Cytoscape (Figure 4). Eight modules of interacting DEGs were predicted.

**FIGURE 5:**
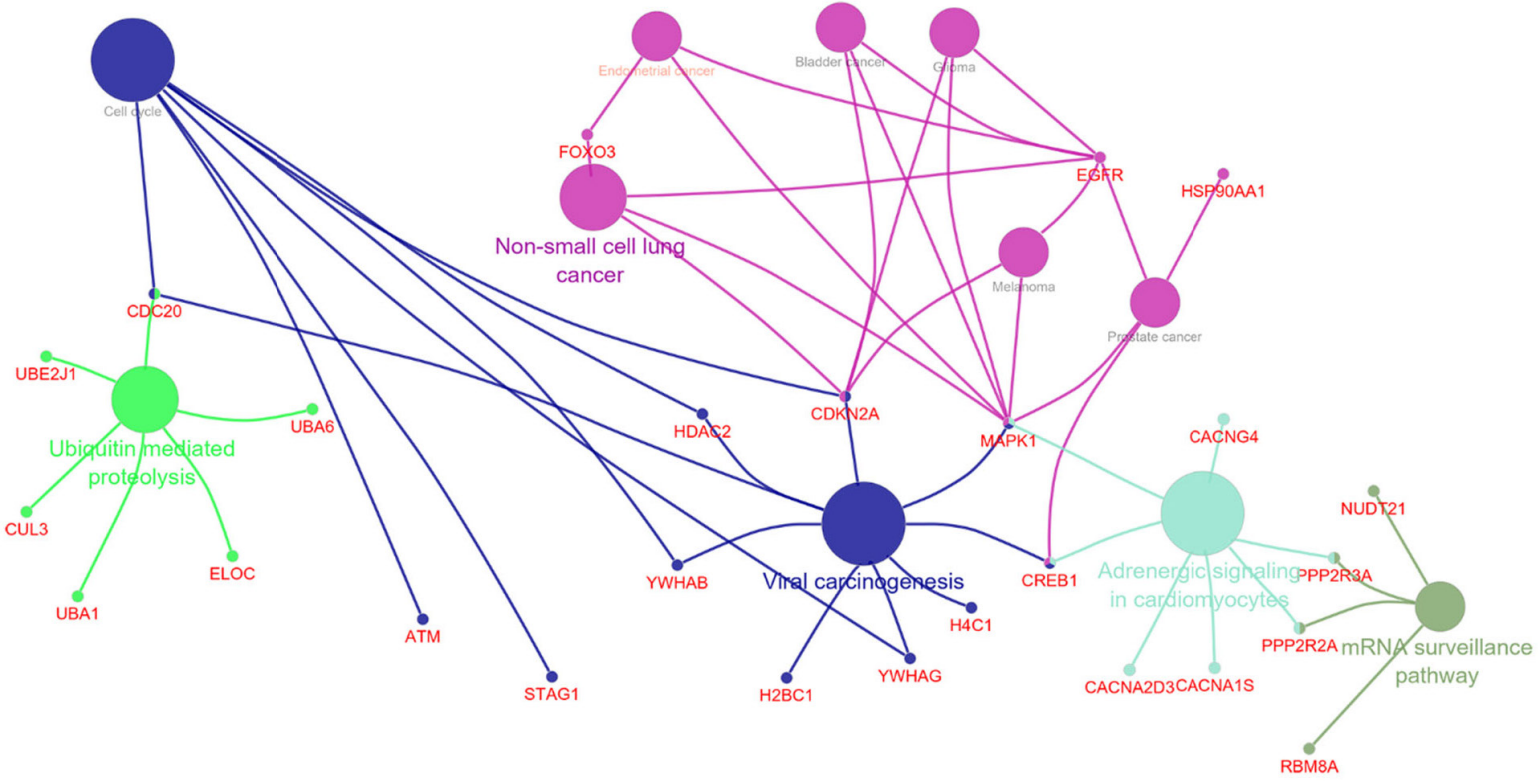
Pathway Enrichment network for DEGs (GSE64951) in top three sub‐clusters of PPI network.

The network modules were constructed by using the criteria; degree cutoff= 2, node score cutoff = 0.2 and K − core = 2.^55^ The score of the module is according to the significance of interaction of genes^56^ that is why we have selected first three modules having higher scores. The topmost module 1 (score 12) consisted of 20 nodes with 13 downregulated and 7 upregulated DEGs connected by 94 edges. The lists of DEGs obtained from 3 modules were imported to ClueGo for pathway enrichment analysis. The DEGs were found to be involved in main five pathways of cancer that is, Viral carcinogenesis, Ubiquitin mediated proteolysis, non‐small lung cancer, Andrenergic signalling in cardiomycytes and mRNA surveillance pathway (Figure 5).

Epidermal growth factor receptor (EGFR) found to be involved in non‐small lung cancer having connected pathways with prostate cancer, melanoma, glioma, bladder cancer and endometrial cancer. Some DEGs were found to be involved in more than one main pathways like MAPK1 and CREB1 were found in viral carcinogenesis, adrenergic signalling and non‐small cell lung cancer. Three DEGs shared the common pathways like CDKN20 in non‐small cell lung cancer and viral carcinogenesis, CDC20 in ubiquitin mediated proteolysis and viral carcinogenesis and PPP2R2A in Adrenergic signalling in cardiomycytes.

#### 3.1.4 Identification of hub genes and their association with other genes in functional network

**FIGURE 6:**
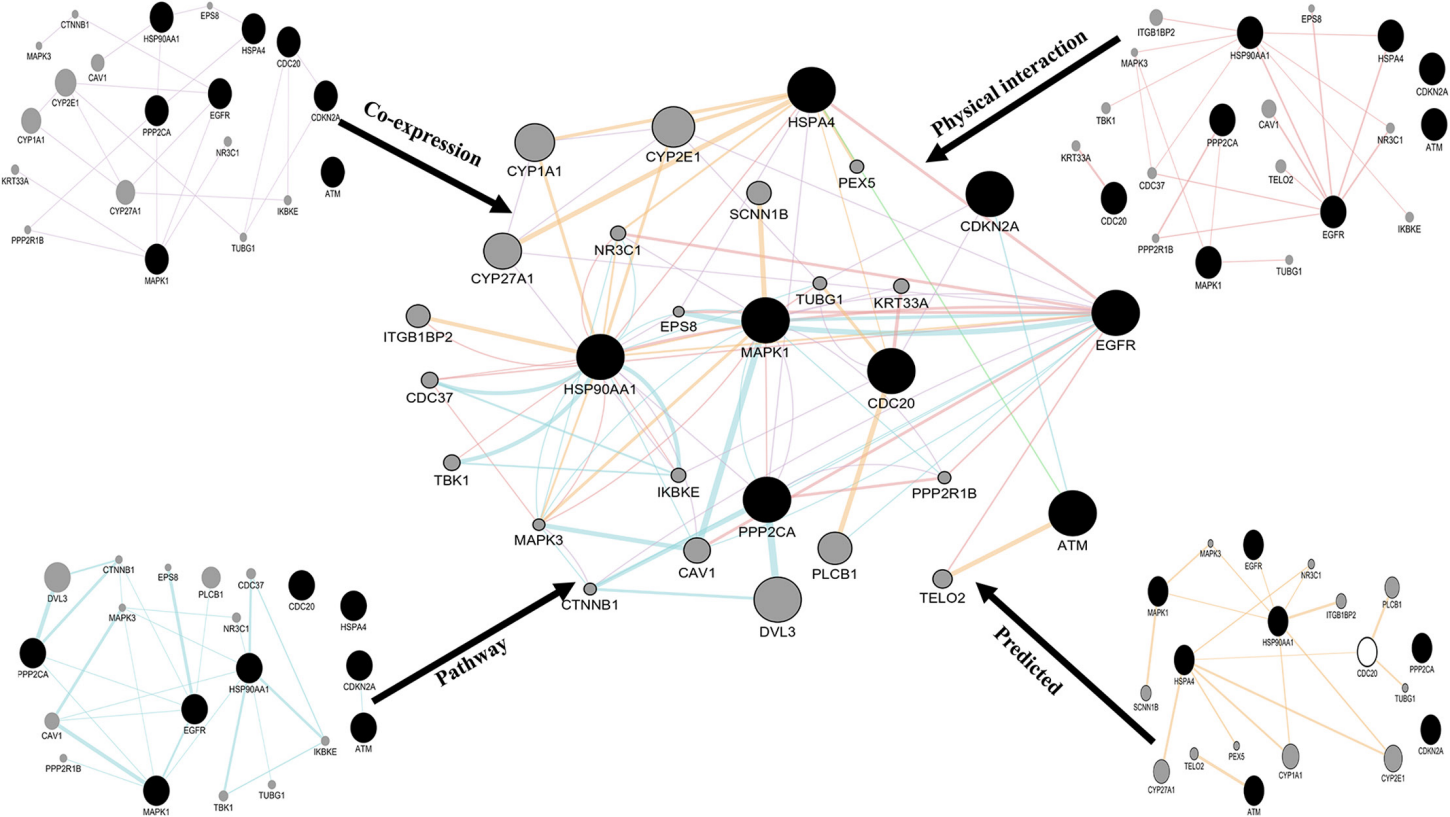
Gene association network of hub genes from gastric cancer DEGs dataset GSE64951. Black nodes = hub genes; Grey nodes = result (associated) genes.

Eight hub genes, EGFR, HSPA90AA1, MAPK1, HSPA4, PPP2CA, CDKN2A, CDC20 and ATM, predicted in more than six methods were selected for further analysis (Table 1). These hub genes were analysed using GeneMANIA, a Cytoscape plug‐in, to construct an interaction network with other human genes. The hub genes are depicted in black, while related genes identified by GeneMANIA are shown in grey. The colours of the edges connecting the nodes (genes) indicate the type of interaction: co‐expression (grey), physical interaction (light pink), predicted (orange), and pathways (light blue) as shown in Figure 6. Nineteen interacting genes were predicted for the eight hub genes. The node sizes in the network represent the enrichment scores of the interacting genes. EGFR is co‐expressed with three genes—CYP27A1, CYP2E1, CTNNB1—and shares pathways with EPS8, CTNNB1, CAV1, and PLCB1. Six genes—EPS8, NR3C1, CAV1, TELO2, CDC37, PPP2R1B—and two hub genes (HSPA4 and HSP90AA1) were predicted to engage in physical interactions. The interaction data for EGFR indicated an exclusive physical interaction with the HSP90AA1 hub gene. Meanwhile, MAPK1 interacts with MAPK3 and SCNN1B, as well as the hub gene HSP90AA1.

**TABLE 1:** Identification of hub genes of gastricQ3 cancer (GSE64951) through 12 methods in CytoHubba.

MCC	DMNC	MNC	Degree	Betweeness	Radiality	Closeness	Stress	Clustering Coefficent	EcCentricity	EPC	Bottle‐Neck	Hub genes	No of methods
CUL3	ZNRF1	EGFR	EGFR	EGFR	EGFR	EGFR	EGFR	NEK7	DDX3X	EGFR	EGFR	**EGFR**	8
TCEB1	ASB12	HSP90AA1	HSP90AA1	HSP90AA1	HSP90AA1	HSP90AA1	HSP90AA1	SERPINB6	POLR2B	HSP90AA1	HSP90AA1	**HSP90AA1**	8
FBX032	FBX032	MAPK1	MAPK1	MAPK1	MAPK1	MAPK1	MAPK1	OSBPL8	HMGB1	MAPK1	MAPK1	**MAPK1**	8
ASB12	ASB10	HSPA4	HSPA4	HSPA4	HSPA4	HSPA4	HSPA4	TBX18	CUL3	HSPA4	HSPA4	**HSPA4**	8
ASB5	FBXL14	PPP2CA	PPP2CA	PPP2CA	PPP2CA	PPP2CA	PPP2CA	TAS2R7	KDM6A	PPP2CA	PPP2CA	**PPP2CA**	8
ASB10	ASB5	CDKN2A	CDKN2A	CDKN2A	CDKN2A	CDKN2A	CDKN2A	OAS1	CDKN2A	CDKN2A	YWHAB	**CDKN2A**	8
FBXL14	UBA6	ATM	ATM	CALM1	ATM	ATM	ATM	CDCP1	KDM6B	ATM	CALM1	**ATM**	6
CDC20	TXNL4A	CDC20	CDC20	CDC20	HDAC2	HDAC2	CALM1	JMJD1C	MYOD1	CDC20	CDC20	**CDC20**	6
UBA1	WHSC1	HDAC2	HDAC2	YWHAB	YWHAB	ATR	RAB7A	ASAP1	SPEN	HDAC2	UBA1		
UBA6	UBE2J1	ATR	ATR	RAB7A 0	PSMC6	PSMC6	YWHAB	GTPBP10	CD24	FOXO3	RAB7A		

#### 3.1.5 mRNA‐miRNA network construction for hub genes

**FIGURE 7:**
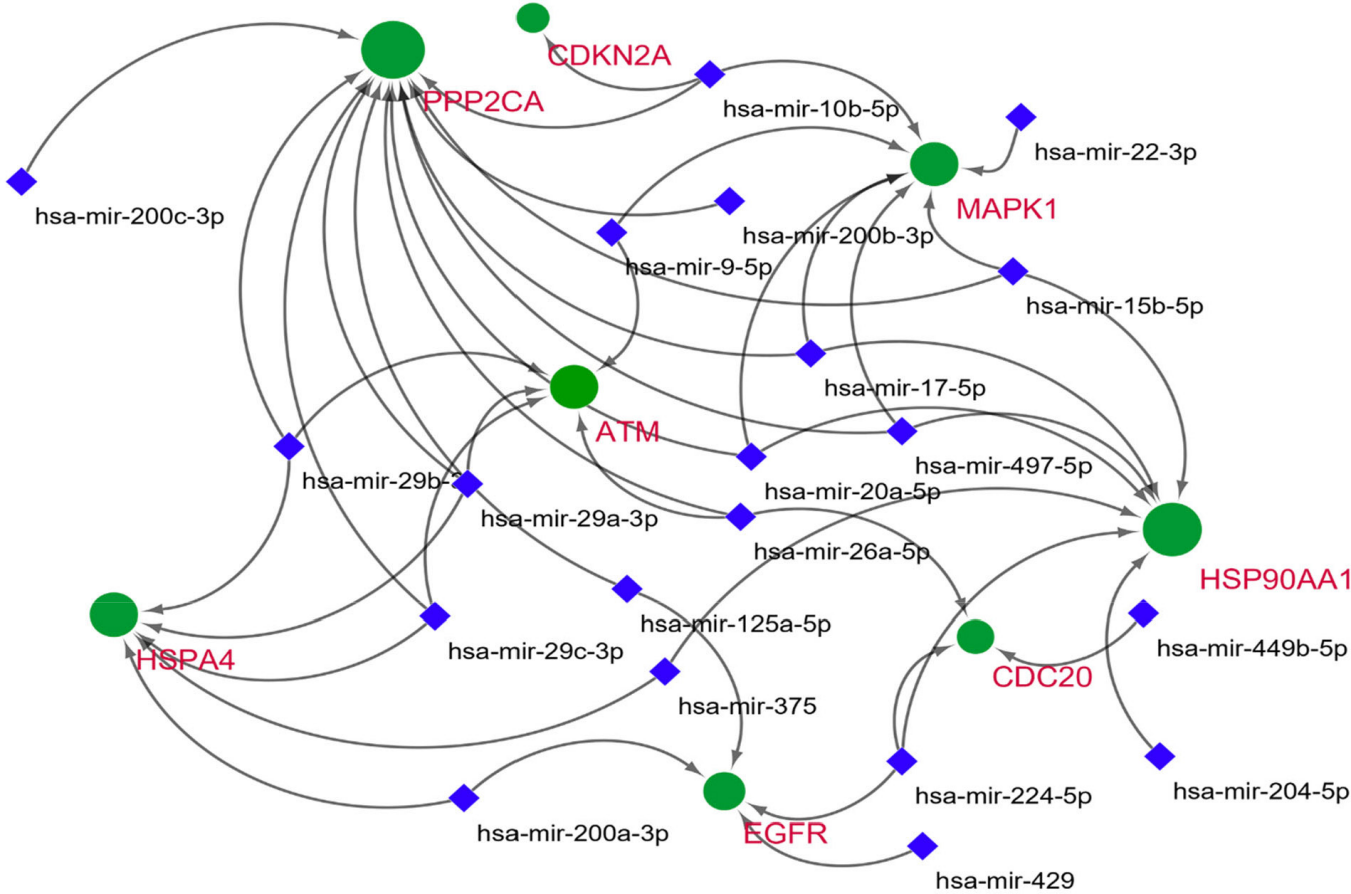
mRNA ‐miRNA network for the hub genes of dataset GSE64951.

The mRNA‐miRNA network generated by mirNet consisted of green rounded nodes for target gene and small blue diamond nodes for miRNA (Figure 7). Total 23 miRNA were predicted for a set of eight target Hub genes.

The highest number (12) of miRNA were predicted for PPP2CA followed by seven for HSP90AA1 and 4 against EFGR. The size of gene node shows the number of related miRNAs, so PPP2CA is the largest nodes in the network while the smallest one is for CDKN2A having only one associated miRNA. The edges are in the form of arrows pointed towards their respective target genes.

#### 3.1.6 Survival analysis of hub genes

The prognostic significance of the hub genes was investigated using the Kaplan–Meier plotter, an online platform^57^ which integrates clinical and expression data for assessing overall survival (OS) of 875 gastric cancer patients.

**FIGURE 8:**
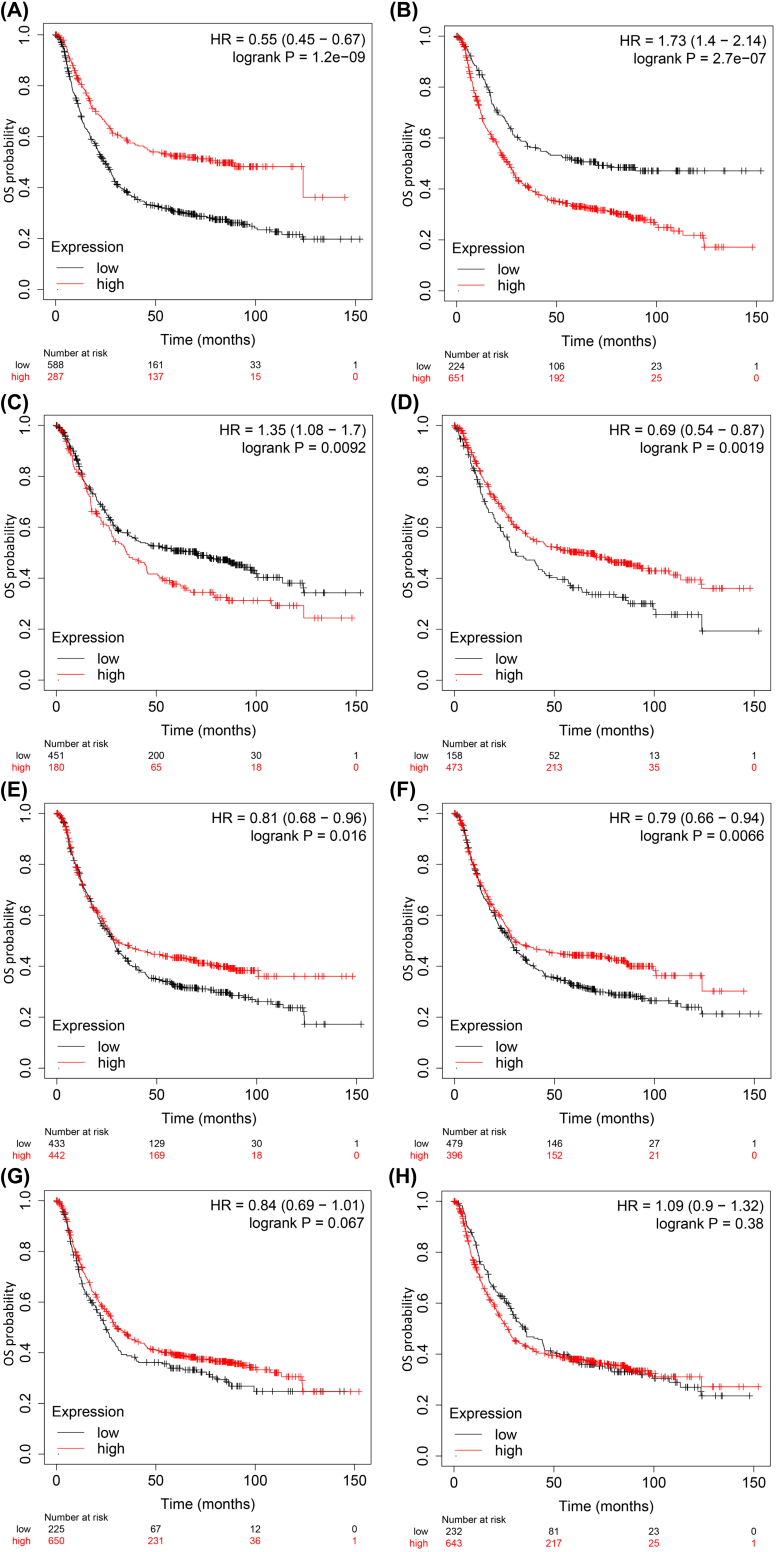
Kaplan–Meier‐Plots for survival analysis of hub genes from the gastric cancer DEGs dataset GSE64951. (A) PP2CA. (B) EGFR. (C) CDKN2A. (D) MAPK1. (E) HSP90AA1. (F) HPSP4. (G) ATM (H) CDC20.

**FIGURE 9:**
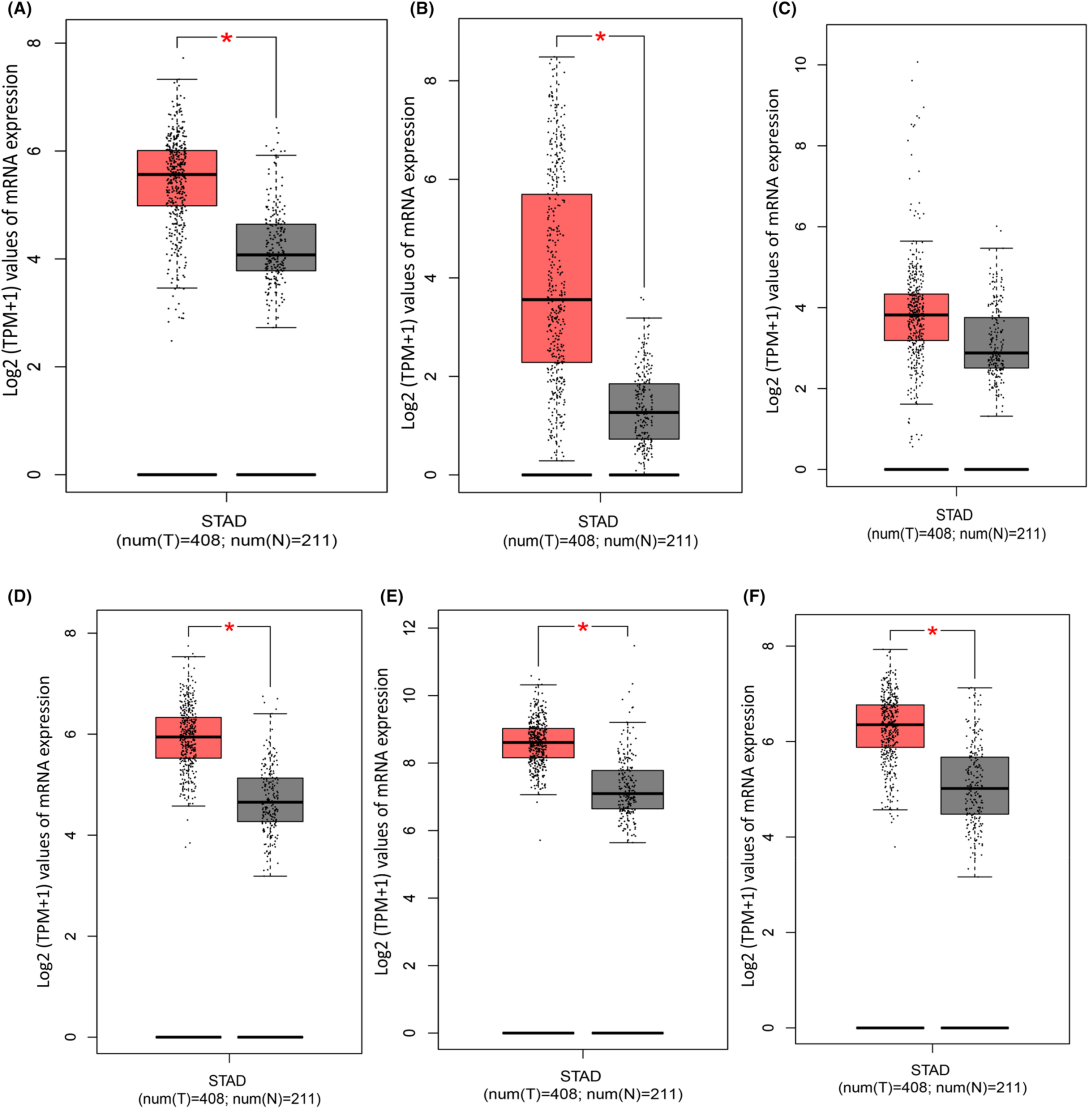
Boxplots for the expression of hub genes in gastric cancer (dataset GSE64951). (A) CDKN2A. (B) EGFR. (C) PP2CA. (D) HSP90AA1. (E) MAPK1. (F) HPSP4. * represents the significant expression status.

The predictive value, for tendency of eight hub genes to affect the cancer severity, was evaluated by using Kaplan–Meier‐Plotter. The relative higher expression of PPP2CA (HR 0.55 [0.45–0.67], *p* = 1.2e^−09^), EGFR (HR:1.73 [1.4–2.14], *p* = 2.7e^−07^), CDKN2A (HR:1.35 [1.08–1.7], *p* = 9.2e^−2^), MAPK1 (HR:0.81[0.68–0.96], *p* = 1.6e^−2^) HSP90AA1 (HR 0.77[0.62–0.96], *p* = 1.8e^−2^) and HPSP4 (HR:0.77[0.62–0.96], *p* = 1.8e^−2^) was related with a worse situation in OS analysis in gastric cancer patients, whereas ATM (HR:0.84 [0.69–1.01], *p* = 6.7e^−2^) CDC20 (HR:1.09 [0.9–1.32], *p* = 3.8e^−3^) had no significant hazard value (*p >* 0.05) (Figure 8A–H). The HR is a measure of how much the hazard (or risk) of an event (such as death or disease progression) changes in one group compared to another. The values in square bracket next HR represents the true risk value range with 95% confidence interval. The *p* value indicates the statistical significance of the HR. The conventional significance level is kept <0.05.^57^ These results showed EGFR as highest risk factor that is, It has highest HR 1.73 with a true range of risk 1.4–2.14 with 95% confidence interval and values are significant with *p* value = 2.7e^−07^ < 0.05. These results were further verified by submitting them to the GEPIA database. The results showed that mRNA levels of five DEGs were significantly (*p* < 0.05) upregulated in gastric cancer samples while only one DEG, PPP2CA, showed not significantly different (*p* > 0.05) expression between affected and normal tissues (Figure 9A–F).

### 3.2 Computer‐aided drug design (CADD)

#### 3.2.1 Selection of receptor protein and ligand molecules

Epidermal growth factor receptor (EGFR) was selected for CADD based on literature review, ^56^, ^58^, ^59^ as the results of survival analysis and pathway enrichment analysis of hub genes. The domain IPRO16245 that is, Tyrosine protein kinase, EGF/ERB/XmrK receptor was found to be involved in the functions like growth, differentiation, metabolism, adhesion, cell death and oncogenesis. The 3D structure of protein EGFR kinase domain (PDB ID: 5CNN) was downloaded from Protein Data Bank (PDB).

The three‐dimensional (3D) structures of 125 marine compounds were downloaded from CMNPD. Only 49 compounds, qualified the drug likeness and non‐toxicity filters, were selected as ligands (Table S2).

#### 3.2.2 Ligand‐receptor molecular docking, molecular dynamics simulation (MDS) and lead identification

The selected chemical compounds were docked with 5CNN by using AutoDock Vina. Eight receptor‐ligand complexes exhibited binding energies ranging from −10.1 to −7.8 kcal/mol and only seven compounds formed hydrogen bonding (Table 2).

**TABLE 2:** Binding energies and perspective interactions of marine life metabolite compounds with 5CNN.

Compounds	CMNPD ID	PubChem CID	Binding affinity (kcal/mol)	Hydrogen bonds	Hydrophobic interactions	2D ligand‐receptor interaction
Adociaquinone A	3305	10364986	−10.1	2	5	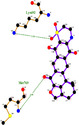
14hydroxymethylxestoquinone	22480	71454859	−9.2	2	4	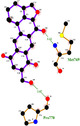
Adociaquinone B	3306	11201041	−8.8	0	7	No interaction
Agelasine	2011	16667745	−8.6	2	9	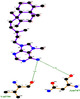
Altersolanol L	24950	42639666	−8.5	5	8	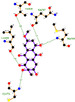
Hirsutanol C	9410	10514702	−8.1	3	6	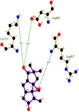
Hippolide A	21383	53262772	−8	1	6	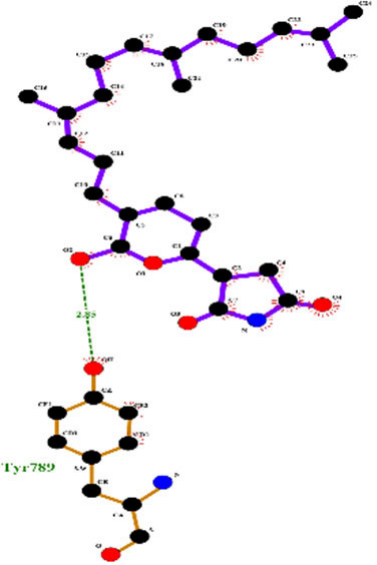
Aspergillide A	18899	70678632	−7.8	2	8	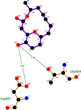

The durability and conformational compatibility of ligand‐receptor complex, over space and time was studied by performing MD Simulation. The top three ligand‐receptor complexes Adociaquinone A‐5CNN, 14hydroxymethylxestoquinone‐5CNN and Agelasine‐5CNN with lowest binding energies were subjected to MD simulation analysis.

**FIGURE 10:**
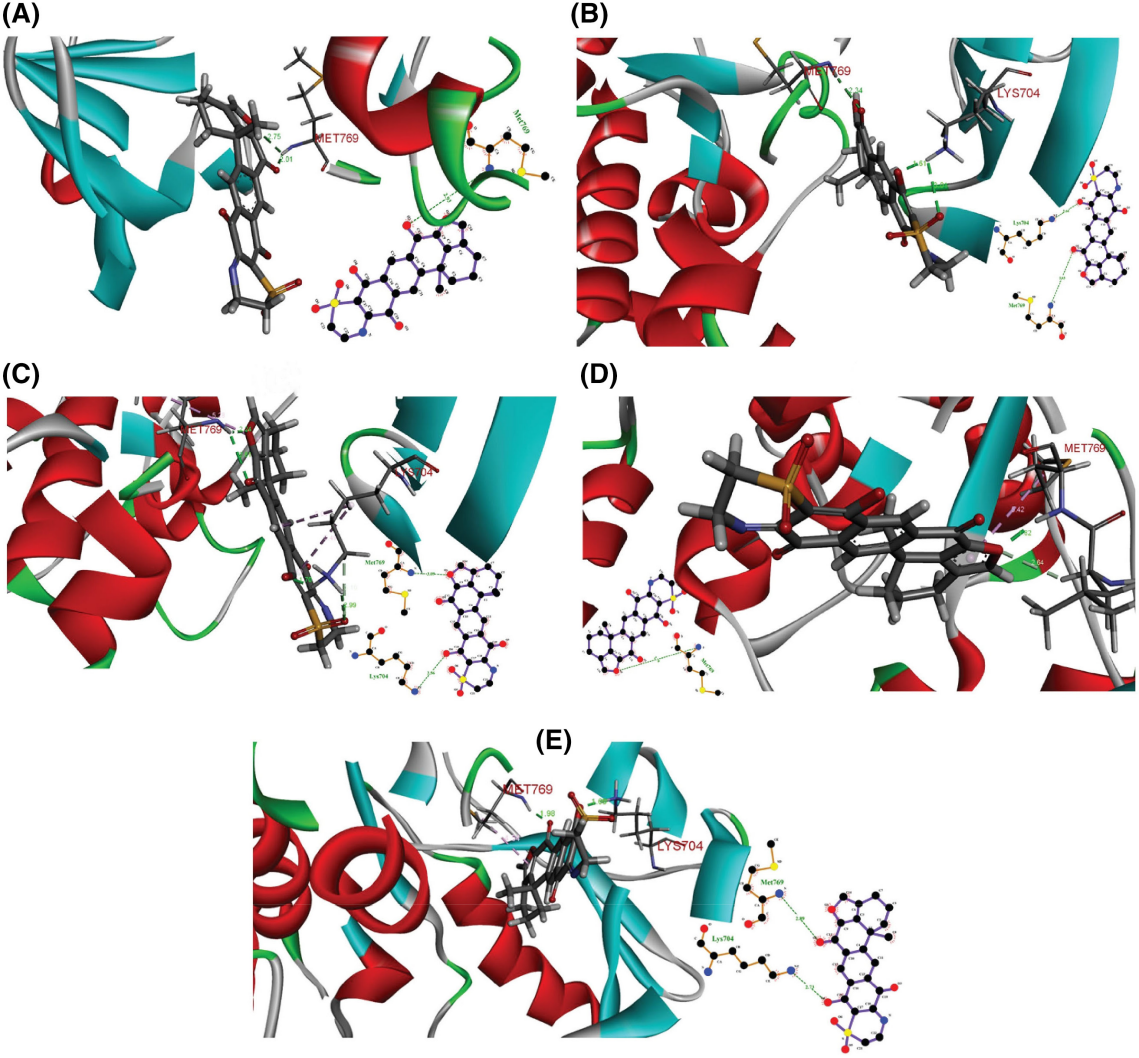
Adociaquinone A‐5CNN 2D & 3D interactions at different poses during Molecular dynamics simulation run at 50 ns. Here, (A) 10 ns, (B) 20 ns, (C) 30 ns, (D) 40 ns, (E) 50 ns.

**FIGURE 11:**
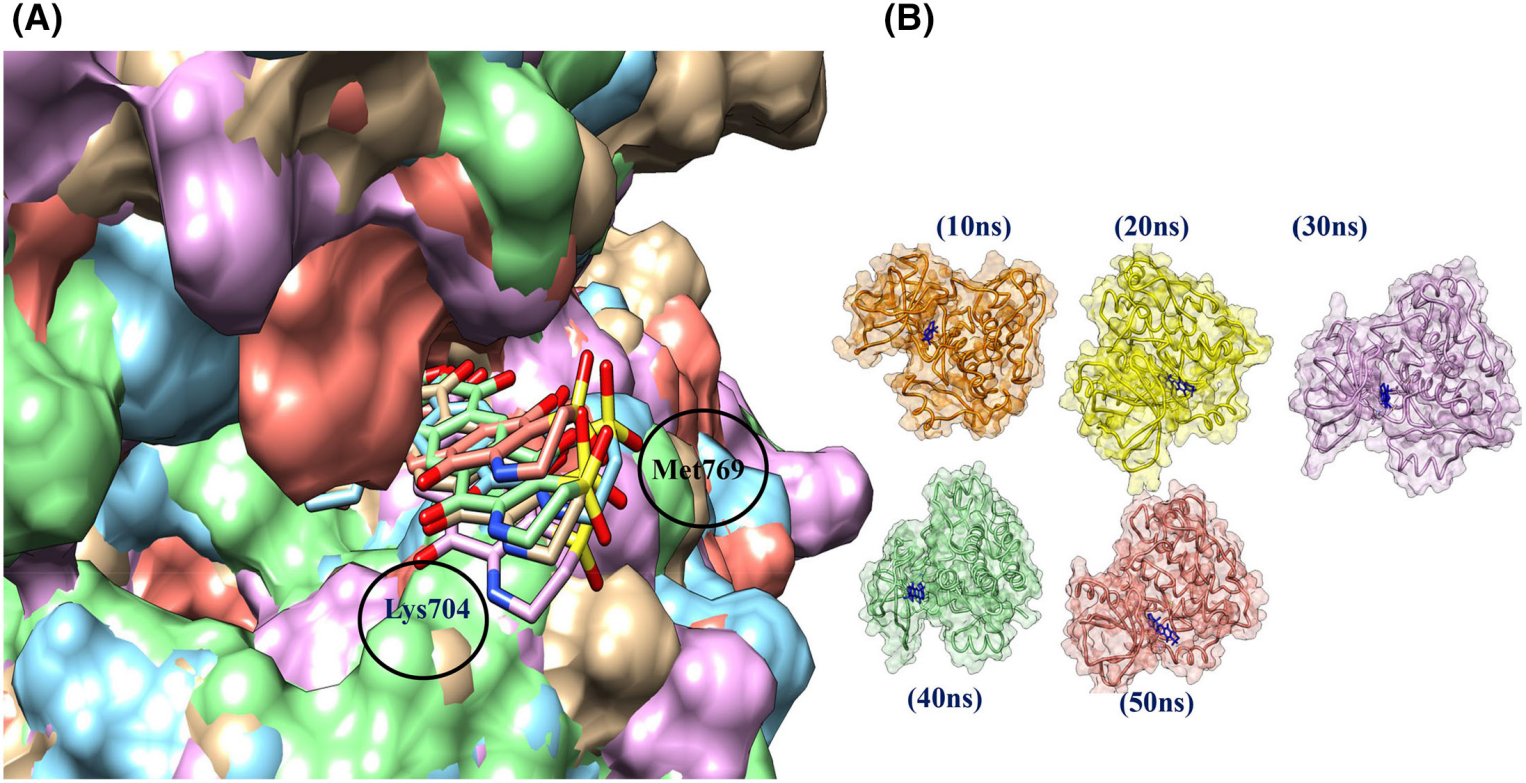
Comparison of different poses of interaction in Adociaquinone A‐5CNN complex during Molecular dynamics simulation run at 50 ns. (A) Superposed poses of complex (B) Tiled poses of complex (ligand in blue).

**FIGURE 12:**
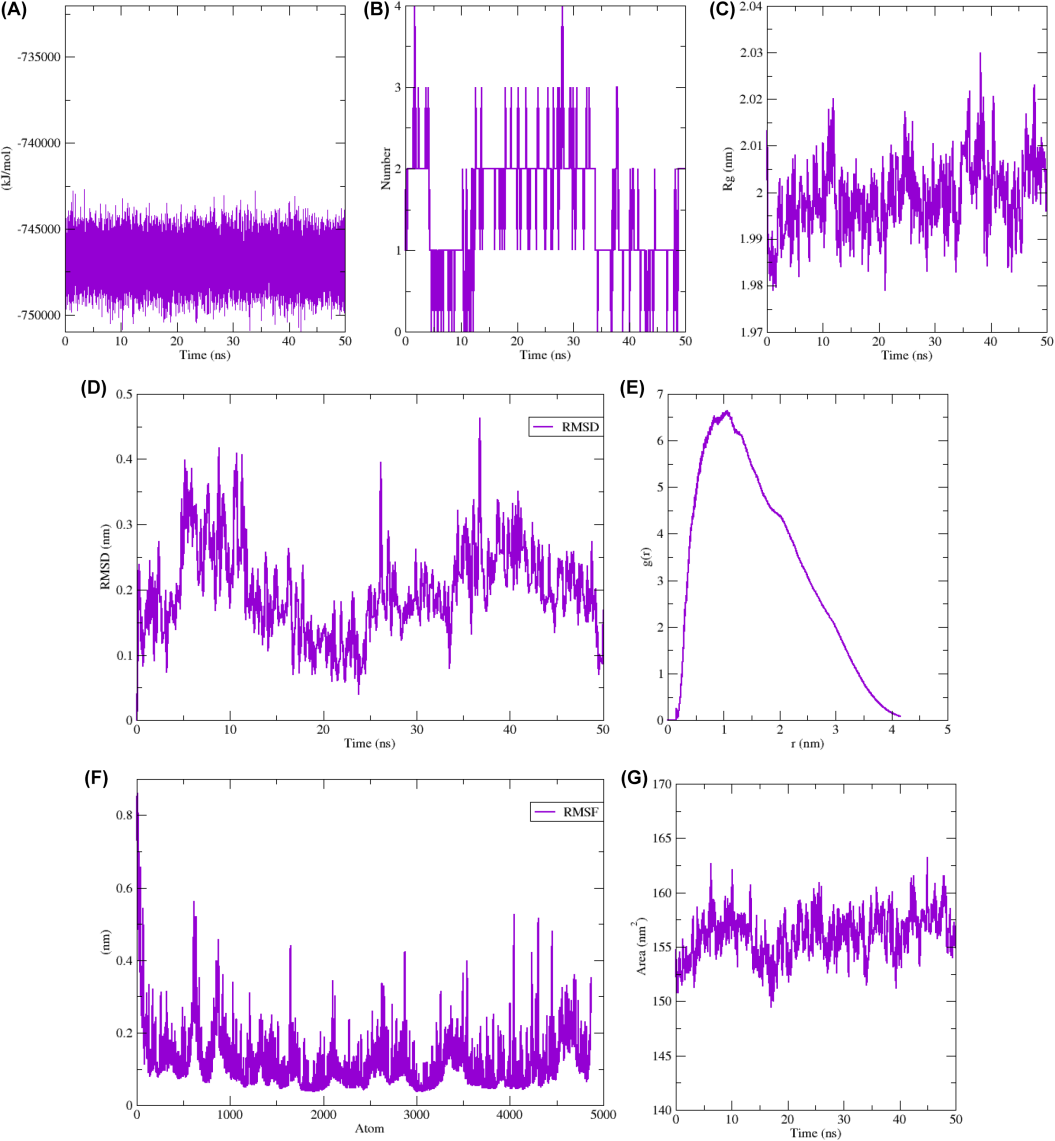
Molecular dynamics simulation graphs run at 50 ns for Adociaquinone A‐5CNN docked complex. (A) Total system energy. (B) Hydrogen bonds. (C) Radius of gyration. (D) Root means square deviation (RMSD). (E) Radial distribution function. (F) Root means square function (RMSF). (G) Solvent accessible surface area.

The Adociaquinone A‐5CNN complex remained stable with minor changes of ligand poses throughout the simulation run. Two hydrogen bonds formed at the time intervals 20, 30 and 50 ns one hydrogen bond was found at 10 and 40 ns (Figure 10A–E). The superposition of all the ligand possesses showed negligible diversion in position of ligand with respect to all time intervals (Figure 11A,B). GROMACS system total energy remained between 745,000 and 750,000kj/mol (Figure 12A). Hydrogen bond formed during simulation time was calculated (Figure 12B). Four hydrogen bonds prevailed in the system. The radius of gyration was 1.98 nm at the start, a peak of 2.5 arose after 25 ns then started dropping down and ending up at 2.5 nm at 50 ns (Figure 12C). A very little drop in radius of gyration throughout the MD simulation run shows stability in the docked complex structure.^55^ Root mean square deviation (RMSD) fluctuated in the start and dropped down after 24 ns then raised at 29 ns and 36 ns then started dropping again (Figure 12D). The radial distribution function (RDF) graph showed a jump of 6.9 g(r) in the start then dropped down suddenly and remained low throughout (Figure 12E). The root mean square fluctuation (RMSF) remained almost constantly low for 4500 atoms peaked for 1.1 nm (Figure 12F). Solvent accessible surface area (SASA) dropped down at19ns but raised after 20 ns (Figure 12G).

Adociaquinone A was identified as a lead compound having binding energy −10.7 kcal/mol with 2 hydrogen bonds that is O6‐Lys692: NZ and C12‐Met769: N with bond distance 3.21 Å and 3.05 Å respectively. It is a lipid extract found in secondary metabolite of the marine sponge *Petrosia alfiani*.

## 4 DISCUSSION

One of the most common tumours worldwide is gastric cancer and it is the third major cause of death in cancer patients.^60^ Maximum number of cases are found in the East Asians regions and one of the leading death causing disease worldwide.^61^ The environmental and genetic factors are main contributing causes of this tumour.^62^, ^63^ This disease can be controlled by early diagnosis and adopting healthy lifestyle. The education and awareness about public health especially regarding cancer leads to early diagnosis which ultimately reduce the death rates.^29^, ^64^, ^65^ Analysis of integrated bioinformatics approaches, which typically concentrate on differentially expressed gene screening, network‐based hub gene discovery and a large amount of survival analysis data, was used to discover the potential biomarkers for diagnosis, therapy and prognosis.^63^, ^66^, ^67^ In the current study 734 DEGs, including 228 downregulated and 507 upregulated genes, were screened from expression dataset GSE64951 retrieved from GEO‐NCBI. The gene ontology analysis has been done by submitting DEGs list to DAVID server. These DEGs were found enriched in GO terms for biological processes that is, cell–cell adhesion, positive regulation of viral process, epidermal cell differentiation, actin cytoskeleton organization, actin filament organization and peptidyl‐serine phosphorylation (PSP). The biological process cell–cell adhesion contained the highest number (26) of the genes with the *p* = 1.05e^−05^. Cell adhesion plays an important role in the maintenance and development of tissues. It is also important for regulation and communication of the cell. Different cell adhesion molecules have been reported to take part in intercellular and cell‐extracellular matrix interactions of cancer.^68^ Cancer progression is a multi‐step process in which some adhesion molecules play a pivotal role in the development of recurrent, invasive and distant metastasis.^69^ Twelve genes have been found to be involved in PSP. In general, serine phosphorylation leads to the formation of multimolecular signalling complexes that regulate diverse biological processes, but in pathological conditions such as tumorigenesis, anomalous phosphorylation may result in the deregulation of some signalling pathways.^70^, ^71^ The highest number of DEGs (73), in molecular function (MF) category, were found to be involved in ATP binding. ATP Binding Cassette (ABC) transporters, widely studied in cancer for their role in drug resistance.^72^ In the last decade however, several studies have established that ABC transporters have additional, fundamental roles in tumour biology, there is strong evidence that these proteins are involved in transporting tumour‐enhancing molecules and/or in PPIs that impact cancer progression and disease prognosis.^73^ The second highest number of DEGs were reported in Poly (A) RNA binding. The ability of proteins to bind to RNA is essential for the development of tumours. The structural and functional diversity of RNA binding proteins (RBPs) is crucial for controlling a number of major cellular activities, including RNA splicing, modification, transport, localization, stability, destruction and translation. The RNA binding proteins maintain the transcriptome through post‐transcriptionally controlling the processing and transportation of RNA, including regulating RNA splicing, polyadenylation, mRNA stability, mRNA localization and translation. Alteration of each process affect the RNA life cycle, produce abnormal protein phenotypes and thus lead to the occurrence and development of tumours.^74^, ^75^ Most of the DEGs were found to be localized in cytosol. The expression of cancer‐causing genes is found to be higher in cytoplasm than other cellular compartments. In a study Kumar et al.^76^ reported significant increase in cytoplasmic and decrease in nuclear expression of S100A2 in oral squamous cell carcinoma in comparison with normal tissues.

PPI network of DEGs was constructed with 937 nodes and 1658 edges, based on the database that is, STRING. Molecular complex detection (MCODE), Cytoscape plug‐in was used for the module construction, to elucidate the biological importance of the gene involved in gastric cancer.^77^ For extracting the closely connected regions with in the protein of interest, MCODE also makes the convenient visualization of the large networks.^78^ The DEGs of selected modules were subjected to ClueGO plugin in Cytoscape for pathway enrichment analysis. Further ClueGO is expanded in CluePedia, which enables detailed analysis of pathways.^79^ It uses the kappa statistics to associate the terms in the network. It generates an interaction network by taking into account the list of genes of interest and their KEGG/and BioCarta pathways.^80^ The pathway network is automatically laid out using the Organic layout algorithm supported by Cytoscape. The GO terms are presented as nodes linked based on a predefined kappa score level and the size of the nodes reflects the enrichment significance of the terms. Kappa score defines the term interactions as the edges of the network and connects the pathways and terms to functional groups on the basis of the shared genes.^44^ All the DEGs have been found to be involved in pathways of five different cancers: viral carcinogenesis, ubiquitin mediated proteolysis, non‐small cell lung cancer (NSCLC), andrenergic signalling in cardiomycytes and 5mRNA surveillance pathway. The occurrence of gastrointestinal metastasis from lung carcinoma is rare but recently Shih‐Chun et al.,^81^ reported a rare case of non‐small cell lung cancer (NSCLC) that initially showed no signs of distant metastasis. However, 5 months after diagnosis, despite receiving treatments for the primary lung cancer, the patient developed gastric metastasis. Viral carcinogenesis is very critical in the case of gastric cancer. The presence of oncogenic viruses in gastric cancer present dual results in response to the treatment, on the one hand, the presence of oncogenic viruses makes the benefits of a successful therapy even greater, but on the other hand, viruses do exist that have a possible therapeutic role in escalating malignancy.^82^

Hub genes are a group of closely related genes that make up a module and are closely related to biological function.^83^ Hub genes were defined as the genes with connectivity (degree) greater than 10 in the genetic interaction network and, incidentally, are the top 10% genes of highest connectivity.^84^ The CytoHubba predicts the hub genes by using 12 statistical methods. These 12 methods are classified into two categories, local and global methods. Local method only evaluates the association of a node with its direct or very closely linked neighbour node that is, Degree, Maximum Neighbourhood Component (MNC), Density of Maximum Neighbourhood Component (DMNC) and Maximal Clique Centrality (MCC). The global method considers the interaction of a node with the other nodes present in the entire network that is, Closeness (Clo), EcCentricity (EC), Radiality (Rad), BottleNeck (BN), Stress (Str), Betweenness (BC) and Edge Percolated Component (EPC).^85^ The selected hub genes HSP90AA1, EFGR, MAPK1, HSPA4, PPP2CA, CDKN2A, CDC20 and ATM, belong to module 1 of PPI network which emphasizes the importance of these genes in gastric cancer. List of hub genes was imported to GeneMANIA for the construction of network showing association with other genes. The network of the query genes with other genes is constructed according to their function and type of interactions.^86^ Moreover, it also identifies the strongly associated genes among the networks and attributes provided on giving a single gene query by using weighing scheme for significance.^87^ The network generated presented different interaction methods of the functional association of key genes or hub genes with the other genes in the network.^88^ The EGFR is a very important gene involved in most of the cancers.^58^, ^89^ It shows three types of interactions with most of the genes that is, co expression (CYP27A1, CYP2E1, CTNNB1), pathways (EPS8, CTNNB1, CAV1, PLCB1), physical interaction (EPS8, NR3C1, CAV1, TELO2, CDC37, PPP2R1B). Our findings have been supported by aforementioned previous studies. CYP27A enzyme is involved in production of 27‐hydroxycholesterol from cholesterol. It has also found to significantly affect the oestrogen receptor positive (ER+) breast cancer and enhance abnormal cell proliferation.^90^ The protein CYP27A1 expression level has been suggested as an important biomarker for postmenopausal breast cancer. Elevated expression of CYP27A1 protein was recently observed to be a marker of late lethal disease in a large cohort of breast cancer patients (Kimbung et al., 2020). The luciferase promotor analysis using RT‐PCR revelled the upregulation of CYP27B1 is controlled by EGF, at translational and transcriptional levels.^91^ A Chip assay generated data revelled the binding of EGF/EGFR complex to the promoter of CYP27B1 in PZ‐HPV7 cells which leads to prostate cancer development.^91^ Kim et al., reported a close association between CTNNB1 and EGFR in lung adenocarcinoma.^92^ They observed the lung adenocarcinoma caused by mutation in CTNNB1 may show post‐operative reoccurrence in patients having mutated EGFR. EGFR pathway substrate 8 (EPS8) is a vital protein which mediates EGFR‐induced activation of Akt and ERK.^93^ It significantly promotes the proliferation by inducing expression of focal adhesion kinase (FAK).^94^, ^95^ It also found to induce resistance in cancer cell lines against chemotherapeutic drugs.^96^

Depending on their target genes, miRNA could function as either oncogene or tumour suppressor under certain circumstances.^97^ The earliest evidence of miRNA involvement in human cancer was provided by Dr. Croce's group from studies attempting to identify tumour suppressors at chromosome 13q 14 regions in B‐cell chronic lymphocytic leukaemia cells. Other studies revealed that miR‐15 and miR‐16‐1 acts as tumour suppressors to induce apoptosis by repressing Bcl‐2, an anti‐apoptotic protein over expressed in malignant non dividing B cells and many solid malignancies.^98^, ^99^ We predicted the associated miRNAs of target hub genes, which would further be analysed for their role in tumour expression or repression. These predicted miRNAs can be used as targets for gastric cancer therapeutics.

A crucial component of medical research is the evaluation of survival after the beginning of a disease or the administration of a medication.^100^ In the best case situation, a straightforward Mann–Whitney test can be used to compare the differential survival of two cohorts.^57^ For escalating the consistency of the results, Kaplan–Meier plotter database was used to confirm the results.^100^ The survival analysis of the hub genes has shown that six of eight hub genes that is, CDKN2A, EGFR, PPP2CA, MAPK1, HSP90AA1 and HPSP4 were significantly correlated with worse overall lethal conditions due to high expression. Epidermal growth factor receptor (EGFR) proved most lethal with highest hazard rate (HR: 1.73). Downstream dimerization and activation of EGFR initiate the processes like cells proliferation, preclusion of apoptosis, angiogenesis induced by tumour, invasion activation and metastatic growth, which can lead to cancer.^101^ In many hostile tumours, the highly expressed genes is EGFR which is associated with tumour growth and invasion.^102^ According to the new studies, EGFR is considered as the main target goal of the gastric cancer therapy.^102^, ^103^, ^104^, ^105^, ^106^ In the current study EGFR has been predicted as an important therapeutic target against gastric cancer.

The tyrosine protein kinase, EGF/ERB/XmrK receptor were identified as the important domain for the EGFR protein. Protein kinases participate in the division, spread, apoptosis and differentiation in a variety of cell processes. EGFR kinase domain (PDB ID: 5CNN) showed 100% query coverage and identity with the sequence of Tyrosine protein kinase, EGF/ERB/XmrK. The 3D structure of 5CNN was downloaded from PDB and used for molecular docking with marine chemical compounds.^107^, ^108^, ^109^, ^110^ The chemical compounds were downloaded from Comprehensive marine natural products database (CMNPD). It is a manually curated database containing more than 34,000 marine organisms derived chemical compounds. The use of natural drug molecules are popularly used now a days to develop safer medicines.^111^ Lower the binding energy of the ligand, greater will be its ability to bind to objective or target.^112^ Adociaquinone A was predicted as a lead compound on the basis of its lower binding energy that is, −10.7 and good stability of interaction with the protein during 50 ns run of MD simulation.^113^, ^114^, ^115^, ^116^, ^117^ In a previous study Adociaquinone‐B was tested against Cdc25B, MKP‐1, and MKP‐3. Cdc25B and MKP‐1 proteins in human cancer cells showed promising results for treating cancer patients. Adociaquinone‐A is the lipid extract of secondary metabolite from the sponge *Petrosia alfiani*. The sponge is bright yellow in colour and becomes brown in the open air.^118^ Sponges are being used as sources of different natural products that can be used in drug development.^119^ A wide variety of natural products, including polyacetylenes, sterols, meroterpenes and alkaloids that are found in Petrosia sponges.^120^ Currently the use of secondary metabolites of sponges is being used in drug development research. Some researchers reported the cytotoxic activities of Petrosia metabolites while most of the studies proved its growth inhibiting activity against human cancer line cells.^121^

## 5 CONCLUSIONS

The mRNA expression profile analysis of gastric (GSE64951) revealed 734 DEGs, among which 507 were up‐regulated and 228 were down‐regulated. Gene ontology studies indicated enrichment in cell–cell adhesion, ATP binding and mRNA binding activities among the DEGs. Notably, eight hub genes—EGFR, HSPA90AA1, MAPK1, HSPA4, PPP2CA, CDKN2A, CDC20, and ATM—were identified as functionally important for further analysis. Among these hub genes, EGFR exhibited the highest expression and HR, making it a prime candidate for targeted therapy against gastric cancer. Using CADD, the marine‐based chemical compound Adociaquinone A showed the most promising binding affinity with EGFR. Additionally molecular dynamics (MD) simulations confirmed the stability of the EGFR‐Adociaquinone A complex over a 50 ns run. Adociaquinone, sourced from the aquatic sponge *Petrosia alfiani*, has been previously reported for its growth‐inhibitory effects on various human cancer cell lines. This suggests its potential as a therapeutic agent against gastric cancer, leveraging EGFR as a druggable target. The findings underscore the significance of marine‐derived compounds in cancer drug discovery and highlight Adociaquinone A as a promising lead molecule for further preclinical and clinical investigations in the treatment of gastric cancer. Moreover predicting microRNA (miRNA) interactions with cancer‐related proteins holds significant medical importance due to its potential impact on cancer diagnosis, prognosis and therapy. Moreover, understanding these interactions facilitates the discovery of novel therapeutic targets and the development of innovative treatment modalities for various cancer types. We predicted the associated miRNAs of target hub genes, 12 for PPP2CA followed by 7 for HSP90AA1 and 4 for EFGR, which would be further analysed for their role in tumour expression or repression. These predicted miRNAs can be used as targets for gastric cancer therapeutics.

## AUTHOR CONTRIBUTIONS

**Mariam Abdulaziz Alkhateeb:** Conceptualization (equal); data curation (equal); formal analysis (equal); investigation (equal); methodology (equal); project administration (equal); software (equal); supervision (equal); validation (equal); writing – review and editing (equal). **Nada H. Aljarba:** Conceptualization (equal); data curation (equal); formal analysis (equal); investigation (equal); supervision (equal); validation (equal); visualization (equal); writing – review and editing (equal). **Qudsia Yousafi:** Conceptualization (equal); data curation (equal); formal analysis (equal); investigation (equal); methodology (equal); project administration (equal); resources (equal); software (equal); writing – original draft (equal); writing – review and editing (equal). **Fatima Anwar:** Conceptualization (equal); data curation (equal); formal analysis (equal); methodology (equal); resources (equal); software (equal); writing – original draft (equal). **Partha Biswas:** Formal analysis (equal); investigation (equal); methodology (equal); project administration (equal); software (equal); supervision (equal); validation (equal); visualization (equal); writing – original draft (equal); writing – review and editing (equal).

## CONFLICT OF INTEREST STATEMENT

The authors declare no conflict of interest.

## Supporting information


**Tables S1‐S2.** Supporting Information.

## Data Availability

All the related to the study data have been included in the study.
