# Supplementary material for: Elucidating gastric cancer mechanisms and therapeutic potential of Adociaquinone A targeting EGFR: A genomic analysis and Computer Aided Drug Design (CADD) approach
Source: J Cell Mol Med. 2024 Oct 21;28(20):e70133. doi: 10.1111/jcmm.70133 (PMC11493557; doi:10.1111/jcmm.70133)
Supplement: Supplementary file 1 — Tables S1‐S2. Supporting Information. [file JCMM-28-e70133-s001.docx]

**Table S1. Marine natural compounds selected from literature survey**

|  | **Marine Compound** | **Source** | **Type** | **CMNPD ID** | **Pubchem ID** | **Calonical Smilies** |
| --- | --- | --- | --- | --- | --- | --- |
| 1 | **Agelasine** | 7,9-dialkylpurinium salts in marine sponges | Alkaloids | CMNPD2011 | 16667745 | CC1CCC2(C(C1(C)CCC(=CC[N+]3=CN(C4=C(N=CN=C43)N)C)C)CCC=C2C)C.[Cl-] |
| 2 | **Fascaplysin** | marine sponge Fascaplysinopsis bergquist sp. |  | CMNPD3288 | 73292 | C1=CC=C2C(=C1)C3=C(N2)C4=[N+](C=C3)C5=CC=CC=C5C4=O.[Cl-] |
| 3 | Zalypsis | tetrahydroisoquinoline alkaloid of marine origin |  | NO RESULT | 16061448 | CC1=CC2=C(C3C4CC5=C(C(=C6C(=C5C(N4C(C(C2)N3C)O)CNC(=O)C=CC7=CC(=CC=C7)C(F)(F)F)OCO6)C)OC(=O)C)C(=C1OC)O |
| 4 | **Bostrycin** | hydroxymethoxytetrahydro-5- methylanthracenedione that was isolated from marine fungi |  | CMNPD17636 | 10042612 | CC1(CC2=C(C(C1O)O)C(=C3C(=O)C=C(C(=O)C3=C2O)OC)O)O |
| 5 | Benzothiazole | marine bacterium Erythrobacter sp |  | **NO RESULT** | 7222 | C1=CC=C2C(=C1)N=CS2 |
| 6 | Apratoxin S4-1e | marine cyanobacteria | Macrolides | **NO RESULT** | 70681359 | CCC(C)C1C(=O)N2CCCC2C(=O)OC(CC(CC(C(C3=NC(CCC(=O)NC(C(=O)N(C(C(=O)N1C)C)C)CC4=CC=C(C=C4)OC)CS3)C)O)C)C(C)(C)C |
| 7 | **Aspergillide A** | fungus Aspergeillus ostianus |  | CMNPD18899 | 70678632 | CC1CCCC=CC2CCC(C(O2)CC(=O)O1)O |
| 8 | Chromomycin SA2 | marine-derived Streptomyces sp |  | **NO RESULT** | 53379616 | CC1C(C(CC(O1)OC2C(CC3=C(C2=O)C(=C4C(=C3)C=C(C(=C4O)C)OC5CC(C(C(O5)C)OC(=O)C)OC6CC(C(C(O6)C)OC)O)O)C(C(=O)O)OC)OC7CC(C(C(O7)C)O)OC8CC(C(C(O8)C)OC(=O)C(C)C)(C)O)O |
| 9 | Lobophorin C | marine sponge-associated actinomycetal strain AZS17 |  | CMNPD20732 | 139587157 | CC1CC(C(C2C1C3(C(C=C2)C(=CCC(C(=CC4C=C(C(CC45C(=O)C(=C3O)C(=O)O5)C)CO)C)OC6CC(C(C(O6)C)NC(=O)OC)(C)N)C)C)OC7CC(C(C(O7)C)O)OC8CC(C(C(O8)C)OC9CC(C(C(O9)C)OC)O)O)C |
| 10 | Lobophorin D | marine sponge-associated actinomycetal strain AZS17 |  | CMNPD20733 | 139584775 | CC1CC(C(C2C1C3(C(C=C2)C(=CCC(C(=CC4C=C(C(CC45C(=O)C(=C3O)C(=O)O5)C)CO)C)OC6CC(C(C(O6)C)NC(=O)OC)(C)N)C)C)OC7CC(C(C(O7)C)O)OC8CC(C(C(O8)C)OC9CC(C(C(O9)C)OC)O)O)C |
| 11 | Spongistatin1 | natural marine compound |  | **NO RESULT** | 9898465 | CC1C2CCCC=CC3CC(CC4(O3)CC(CC(O4)CC(=O)C(C(C(C(=C)CC5CC(CC6(O5)CC(CC(O6)CC(=O)OC7C(C(C(C(O2)(CC1O)O)O)OC(C7O)CC(=C)CC(C=CC(=C)Cl)O)C)OC(=O)C)(C)O)C)OC(=O)C)C)OC)O |
| 12 | **Acetylapoaranotin** | marine Aspergillus sp | Peptides | **NO RESULT** | 9826799 | CC(=O)OC1C=CC=C2C1N3C(=O)C45CC6=COC=CC(C6N4C(=O)C3(C2)SS5)OC(=O)C |
| 13 | **Hemiasterlin** | marine sponges |  | CMNPD8172 | 5352092 | CC(C)C(C=C(C)C(=O)O)N(C)C(=O)C(C(C)(C)C)NC(=O)C(C(C)(C)C1=CN(C2=CC=CC=C21)C)NC |
| 14 | **Jasplakinolide V** | marine sponge Jaspis splendens |  | CMNPD21256 | 52937878 | CC1CC(OC(=O)CC(NC(=O)C(N(C(=O)C(NC(=O)C(CC(=C1)C)C)C)C)CC2=C(NC3=CC=CC=C32)Br)C4=CC(=C(C=C4)O)O)C |
| 15 | Lagunamide C | Lyngbya majuscula, a marine cyanobacterium |  | CMNPD21034 | 56839938 | CCC(C)C1CC(C(CC=C(C(=O)OC(C(=O)NC(C(=O)N(C(C(=O)N(CC(=O)NC(C(=O)N(C(C(=O)O1)C)C)C(C)CC)C)CC2=CC=CC=C2)C)C)C(C)CC)C)O)C |
| 16 | Largazole | marine cyanobacterium Symploca sp |  | CMNPD17731 | 24757913 | CCCCCCCC(=O)SCCC=CC1CC(=O)NCC2=NC(=CS2)C3=NC(CS3)(C(=O)NC(C(=O)O1)C(C)C)C |
| 17 | **Nocardioazine A** | bacterium Nocardiopsis sp. (CMB-M0232) |  | **NO RESULT** | 53307013 | CC12CC3C(=O)N4C5CC6(C4N(C7=CC=CC=C76)C)CC8C(O8)(CN(C1N3C5=O)C9=CC=CC=C29)C |
| 18 | Pardaxin | Red sea Moses sole (a small fish) |  | **NO RESULT** | 16143997 | CCC(C)C(C(=O)NC(C(C)CC)C(=O)NC(CO)C(=O)NC(CO)C(=O)N1CCCC1C(=O)NC(CC(C)C)C(=O)NC(CC2=CC=CC=C2)C(=O)NC(CCCCN)C(=O)NC(C(C)O)C(=O)NC(CC(C)C)C(=O)NC(CC(C)C)C(=O)NC(CO)C(=O)NC(C)C(=O)NC(C(C)C)C(=O)NCC(=O)NC(CO)C(=O)NC(C)C(=O)NC(CC(C)C)C(=O)NC(CO)C(=O)NC(CO)C(=O)NC(CO)C(=O)NCC(=O)NCC(=O)NC(CCC(=O)N)C(=O)NC(CCC(=O)O)C(=O)O)NC(=O)C(CCCCN)NC(=O)C3CCCN3C(=O)C(C(C)CC)NC(=O)C(CC(C)C)NC(=O)C(C)NC(=O)C(CC4=CC=CC=C4)NC(=O)C(CC5=CC=CC=C5)NC(=O)CN |
| 19 | Rhizochalin | marine sponge Rhizochalina incrustata | sphingolipid | CMNPD3742 | 44445587 | CC(C(CCCCCCCC(=O)CCCCCCCCCCCCCCC(C(C)N)OC1C(C(C(C(O1)CO)O)O)O)O)N |
| 20 | **Methyl spongoate (MESP)** | Sanya soft coral Spongodes sp | Steroids | **NO RESULT** | 46179749 | CC(C)CCCC(C1CCC2C1(CCC3C2CCC4C3(C=CC(=O)C4)C)C)C(=O)OC |
| 21 | Dieckol(Tannins) | marine brown alga Ecklonia cava. |  | CMNPD1845 | 3008868 | C1=C(C=C(C=C1O)OC2=C(C=C(C3=C2OC4=C(C=C(C=C4O3)OC5=C(C=C(C=C5O)OC6=C(C=C(C7=C6OC8=C(C=C(C=C8O7)O)O)O)O)O)O)O)O)O |
| 22 | Astaxanthin | red-orange colored carotenoid from marine origin | Terpenes/Terpenoids | CMNPD7249 | 5281224 | CC1=C(C(CC(C1=O)O)(C)C)C=CC(=CC=CC(=CC=CC=C(C)C=CC=C(C)C=CC2=C(C(=O)C(CC2(C)C)O)C)C)C |
| 23 | Culobophylin A | cultured soft coral Lobophytum crassum |  | CMNPD21567 | not found |  |
| 24 | **Hippolide A** | marine sponge Hippospongia lachne |  | CMNPD21383 | 53262772 | CC(=CCCC(=CCCC(=CCCC1=CCC(OC1O)C2CC(=O)NC2=O)C)C)C |
| 25 | **Irciformonin B** | marine sponge Ircinia sp |  | CMNPD21381 | 54669792 | CC(=CC(CC(=CCCC1=COC=C1)C)O)CCC(C2(CCC(=O)O2)C)O |
| 26 | **15-acetylirciformonin B** | marine sponge Ircinia sp |  | CMNPD21379 | 54669794 | CC(=CC(CC(=CCCC1=COC=C1)C)O)CCC(C2(CCC(=O)O2)C)OC(=O)C |
| 27 | **10-acetylirciformonin B** | marine sponge Ircinia sp |  | CMNPD21380 | 54669795 | CC(=CC(CC(=CCCC1=COC=C1)C)OC(=O)C)CCC(C2(CCC(=O)O2)C)O |
| 28 | **Irciformonin F** | marine sponge Ircinia sp |  | CMNPD19224 | 54669793 | CC(=CC(CC(=CCCC1=COC=C1)C)OC(=O)C)CCC(C2(CCC(=O)O2)C)OC(=O)C |
| 29 | **Sarcophine** | Red Sea soft coral Sarcophyton glaucum. |  | CMNPD136 | 6436805 | CC1=CCCC2(C(O2)CCC(=CC3C(=C(C(=O)O3)C)CC1)C)C |
| 30 | **Siphonaxanthin** | siphonaceous green algae Codium fragile |  | CMNPD12509 | 5380124 | CC1=C(C(CC(C1)O)(C)C)CC(=O)C(=CC=CC(=CC=CC=C(C)C=CC=C(C)C=CC2C(=CC(CC2(C)C)O)C)C)CO |
| 31 | **Smenospongine** | Indonesian marine sponge Dactylospongia elegans |  | CMNPD3299 | 3081931 | CC1CCC2(C(C1(C)CC3=C(C(=CC(=O)C3=O)N)O)CCCC2=C)C |
| 32 | **Waixenicin A** | marine soft coral Sarcothelia edmondsoni |  | CMNPD2041 | 73755210 | CC1=CCCC(=C)C2C(CC1)C(=COC2OC(=O)C)C(CC=C(C)COC(=O)C)OC(=O)C |
| 33 | **Agelasine B** | marine sponge Agelas clathrodes |  | CMNPD2012 | 6439899 | CC1CCC2(C(C1(C)CCC(=CCN3C=[N+](C4=NC=NC(=C43)N)C)C)CCC=C2C)C |
| 34 | **Granulatimide** | ascidian Didemnum granulatum |  | CMNPD10016 | 5324595 | C1=CC=C2C(=C1)C3=C4C(=C5C(=C3N2)NC=N5)C(=O)NC4=O |
| 35 | **Isogranulatimide** | ascidian Didemnum granulatum | alkaloids | CMNPD10017 | 135418335 | C1=CC=C2C(=C1)C3=C4C(=C(NC4=O)O)C5=CN=CN5C3=N2 |
| 36 | **Hyrtioreticulins B** | marine sponge Hyrtios reticulatus |  | **NO RESULT** | 70684035 | C1C(NC(C2=C1C3=C(N2)C=CC(=C3)O)CC4=CN=CN4)C(=O)O |
| 37 | **Hyrtioreticulins A** | marine sponge Hyrtios reticulatus |  | **NO RESULT** | 70694527 | C1C(NC(C2=C1C3=C(N2)C=CC(=C3)O)CC4=CN=CN4)C(=O)O |
| 38 | **Lamellarin D** | marine organisms, such as mollusks, ascidians, and sponges |  | CMNPD2477 | 9892144 | COC1=C(C=CC(=C1)C2=C3C4=CC(=C(C=C4C=CN3C5=C2C6=CC(=C(C=C6OC5=O)O)OC)O)OC)O |
| 39 | **5-(2,4-Dimethylbenzyl) pyrrolidin-2-one (DMBPO)** | marine Streptomyces VITSVK5 spp. | Amine Derivatives | **NO RESULT** | 104687068 | CC1=CC(=C(C=C1)CC2CCC(=O)N2)C |
| 40 | Bryostatin1 | invertebrate marine bryozoan Bugula neritina |  | CMNPD5152 | 5280757 | CCCC=CC=CC(=O)OC1C(=CC(=O)OC)CC2CC(OC(=O)CC(CC3CC(C(C(O3)(CC4CC(=CC(=O)OC)CC(O4)C=CC(C1(O2)O)(C)C)O)(C)C)OC(=O)C)O)C(C)O |
| 41 | Eribulin | marine sponge Halichondria okadai |  | **NO RESULT** | 11354606 | CC1CC2CCC3C(=C)CC(O3)CCC45CC6C(O4)C7C(O6)C(O5)C8C(O7)CCC(O8)CC(=O)CC9C(CC(C1=C)O2)OC(C9OC)CC(CN)O |
| 42 | Halichoblelide A | strain of Streptomyces seperated from marine fish Halichoeres bleekeri |  | **NO RESULT** | 102024100 | CCC1C(OC(C1OC2CC(C(C(O2)C)O)O)OC(=O)C(C)C(C(C)C3C(C=CC=CC(=O)OC(C(C=CC=CC(=O)O3)C)C(C)C(C(C)C4(CC(C(C(O4)C)CC)OC5CC(C(C(O5)C)O)O)O)O)C)O)C |
| 43 | Halichoblelide B | strain of Streptomyces seperated from marine fish Halichoeres bleekeri |  | CMNPD21934 | 60144763 | CCC1C(OC(CC1OC)(C(C)C(C(C)C2C(C=CC=CC(=O)OC(C(C=CC=CC(=O)O2)C)C(C)C(C(C)C3(CC(C(C(O3)C)CC)OC4CC(C(C(O4)C)O)O)OC)O)C)O)OC)C |
| 44 | Halichoblelide C | strain of Streptomyces seperated from marine fish Halichoeres bleekeri |  | CMNPD21935 | 60144915 | CCC1C(OC(CC1OC)(C(C)C(C(C)C2C(C=CC=CC(=O)OC(C(C=CC=CC(=O)O2)C)C(C)C(C(C)C3(CC(C(C(O3)C)C)OC4CC(C(C(O4)C)O)O)OC)O)C)O)OC)C |
| 45 | **Laulimalide** | marine sponges Mycale hentscheli and Cacospongia mycofijiense |  | CMNPD8162 | 6918457 | CC1CC2CC=CC(O2)CC=CC(=O)OC(CC3C(O3)C(CC(=C)C1)O)C(C=CC4CC(=CCO4)C)O |
| 46 | **Peloruside A** | marine sponges Mycale hentscheli and Cacospongia mycofijiense |  | CMNPD11091 | 6918506 | CCC(CO)C=C(C)C1CC(CC(C(C2(C(C(CC(O2)CC(C(C(=O)O1)O)OC)OC)O)O)(C)C)O)OC |
| 47 | **Mycoepoxydiene** | first isolated from the fermentation broth of OS-F66617, a fungal strain obtained from the deadwood of forests in Brazil [45]. Lin et al. [45] isolated MED from the marine fungus Diaporthe sp |  | **NO RESULT** | 11300750 | CC1C2C=CC=CC(C1C3C(C=CC(=O)O3)OC(=O)C)O2 |
| 48 | Salarin C | Fascaplysinopsis sp., a marine sponge |  | CMNPD19099 | 24867082 | CCCCCCCC(=O)OCCC=CC1C(O1)C2C(C3C(O3)CCC(=CC4=NC(=C(O4)C)C=CC=CC(=O)O2)C)OC(=O)NC(=O)C |
| 49 | Salarin A | Fascaplysinopsis sp., a marine sponge |  | CMNPD19100 | 24766296 | CCCCCCCC(=O)OCCC=CC1C(O1)C2C(C3C(O3)CCC(=CC(=O)N(C(=O)C=CC=CC(=O)O2)C(=O)C)C)OC(=O)NC(=O)C |
| 50 | Spirastrellolide A | marine sponge Spirastrella coccinea |  | CMNPD16906 | 70690672 | CC1CCC2C(C(CC3(O2)CCC(C4(O3)CC(C(O4)CC=CCC=CCC(C(=O)O)O)OC(=O)CC5CCCC(O5)CC(CC(CC6C(C=CC7(O6)CCC(C(O7)C(C1O)O)OC)C)O)O)C)OC)Cl |
| 51 | Spirastrellolide B | ''''''''''''''''''''''''''''''''''''''''''''''''''''''''''''''''''''''''' |  | CMNPD16900 | 70696901 | CC1CCC2CC(CC3(O2)CCC(C4(O3)CC(C(O4)CC=CCC=CCC(C(=O)O)O)OC(=O)CC5CCCC(O5)CC(CC(CC6C(CCC7(O6)CCC(C(O7)C(C1O)O)OC)C)O)O)C)OC |
| 52 | Neamphamide B | Australian sponge Neamphius huxleyi | Peptides/Polypeptides | CMNPD22496 | 70696602 | CC1C(C(=O)NC(C(=O)NC(C(=O)NC(C(=O)N(C(C(=O)NC(C(=O)N2CCCCC2C(=O)O1)C(C3=CC=C(C=C3)O)OC)CCC(=O)N)C)CC(C)C)CCCN=C(N)N)C(C)O)NC(=O)C(C(C)C(C)C(=O)N)NC(=O)C(C(C(CCCN=C(N)N)NC(=O)C(CC(=O)N)NC(=O)C(C)C(C(C)CC(C)C)O)O)O |
| 53 | Viequeamide A | “button” cyanobacterium (Rivularia sp.) |  | CMNPD22296 | 71466917 | CCC(C)C1C(=O)N2CCCC2C(=O)N(C(C(=O)NC(C(=O)OC(C(C(=O)NC(C(=O)N(C(C(=O)O1)C(C)C)C)C(C)C)(C)C)CCCC#C)C(C)O)C(C)C)C |
| 54 | Kulokekahilide 2 | Hawaiian marine mollusk Philinopsis speciosa |  | CMNPD18424 | 11491350 | CCC(C)C1C(=O)NC(C(=O)OC(C(C(CC=C(C(=O)OC(C(=O)NC(C(=O)N(C(C(=O)N(CC(=O)N1)C)CC2=CC=CC=C2)C)C)CC(C)C)C)O)C)C(=CC)C)C |
| 55 | Lagunamide A | filamentous marine cyanobacterium, Lyngbya majuscula, |  | CMNPD19915 | 50901239 | CCC(C)C1C(C(CC=C(C(=O)OC(C(=O)NC(C(=O)N(C(C(=O)N(CC(=O)NC(C(=O)N(C(C(=O)O1)C)C)C(C)CC)C)CC2=CC=CC=C2)C)C)C(C)CC)C)O)C |
| 56 | Lagunamide B | '''''''''''''''''''''''''''''''''''''''''''''''''''''''''''''''''''''''''''''''''''''''''''''''''''''''''''''''''' |  | CMNPD19916 | 50901240 | CCC(C)C1C(=O)N(C(C(=O)OC(C(C(CC=C(C(=O)OC(C(=O)NC(C(=O)N(C(C(=O)N(CC(=O)N1)C)CC2=CC=CC=C2)C)C)C(C)CC)C)O)C)C(=CC)C)C)C |
| 57 | cordyheptapeptide C | marine-derived fungus Acremonium persicinum SCSIO 115 |  | CMNPD21991 | 60155059 | CC(C)CC1C(=O)N(C(C(=O)N2CCCC2C(=O)N(CC(=O)NC(C(=O)N(C(C(=O)NC(C(=O)N1)C(C)C)CC3=CC=C(C=C3)O)C)CC4=CC=CC=C4)C)CC5=CC=CC=C5)C |
| 58 | cordyheptapeptide D |  |  | CMNPD21992 | 60155154 | CC(C)CC1C(=O)N(C(C(=O)N2CCCC2C(=O)N(CC(=O)NC(C(=O)N(C(C(=O)NC(C(=O)N1)C(C)C)CC3=CC=C(C=C3)O)C)CC4=CC=CC=C4)C)CC5=CC=C(C=C5)O)C |
| 59 | cordyheptapeptide E |  |  | CMNPD21993 | 60155155 | CCC(C)C1C(=O)NC(C(=O)N(C(C(=O)N2CCCC2C(=O)N(CC(=O)NC(C(=O)N(C(C(=O)N1)CC3=CC=C(C=C3)O)C)CC4=CC=CC=C4)C)CC5=CC=C(C=C5)O)C)CC(C)C |
| 60 | Hoiamide D | both its acid and carboxylate forms of cyanobacterium Symploca sp |  | CMNPD22300 | 56835050 | CCCC(C)C(C(C)C(C(C)C(CC1=NC(=CS1)C2=NC(CS2)(C)C3=NC(CS3)(C)C(=O)NC(C(C)CC)C(C(C)C(=O)O)O)OC)O)O |
| 61 | **Protuboxepin A** | marine-derived fungus Aspergillus sp. SF-5044 |  | **NO RESULT** | 53355697 | CCC(C)C1C2=NC3=C(C=CC=CO3)C(=O)N2C(C(=O)N1)CC4=CC=CC=C4 |
| 62 | **Aeroplysinin 1** | marine sponge Aplysina aerophoba | Phenols/Polyphenols | CMNPD998 | 100308 | COC1=C(C(C(C=C1Br)(CC#N)O)O)Br |
| 63 | Grincamycin B | Streptomyces lusitanus SCSIO LR32 |  | CMNPD21936 | 57332649 | CC1C(CCC(O1)OC2C(OC(CC2O)C3=C(C4=C(C=C3)C(=O)C5=C(C4=O)C=CC(=C5O)CC(C)(CC(=O)O)OC6CCC(C(O6)C)OC7CCC(=O)C(O7)C)O)C)OC8CCC(=O)C(O8)C |
| 64 | Grincamycin C | Streptomyces lusitanus SCSIO LR32 |  | CMNPD21937 | 57332650 | CC1C(CCC(O1)OC2C(OC(CC2O)C3=C(C4=C(C=C3)C(=O)C5=C(C4=O)C=CC(=C5O)CC(C)(CC(=O)O)O)O)C)OC6CCC(=O)C(O6) |
| 65 | Grincamycin D | Streptomyces lusitanus SCSIO LR32 |  | CMNPD21938 | 57332778 | CC1C(CCC(O1)OC(C)(CC2=C(C3=C(C=C2)C(=O)C4=C(C3=O)C=CC(=C4O)C5CC6C(C(O5)C)OC7C(O6)CC(=O)C(O7)C)O)CC(=O)O)OC8CCC(=O)C(O8)C |
| 66 | Grincamycin E | Streptomyces lusitanus SCSIO LR32 |  | CMNPD21939 | 57332779 | CC1C(CCC(O1)OC2C(OC(CC2O)C3=C(C4=C(C=C3)C(=O)C5=C(C4=O)C=C6C(=C5O)CC(CC6=O)(C)OC7CCC(C(O7)C)OC8CCC(=O)C(O8)C)O)C)OC9CCC(=O)C(O9)C |
| 67 | Grincamycin F | Streptomyces lusitanus SCSIO LR32 |  | CMNPD21940 | 57332780 | CC1C(CCC(O1)OC2CC(OC(C2O)C)C3=CC4=C(C(=O)OC5=C4C(=C3O)C(=O)C6=C5C7(C(=O)CC(CC7(C=C6)O)(C)OC8CCC(C(O8)C)OC9CCC(=O)C(O9)C)O)C1=CC=C(C=C1)O)OC1CCC(=O)C(O1)C |
| 68 | **tetrahydroaltersolanol B** | Alternaria sp. ZJ-2008003, a fungus |  | **NO RESULT** | 70683135 | CC1(CC2C(CC1O)C(=O)C3=C(C2O)C=C(C=C3O)OC)O |
| 69 | **Alterporriol L** | mangrove endophytic fungus, Alternaria sp. ZJ9-6B |  | CMNPD20946 | 139587993 | CC1=CC2=C(C=C1O)C(=O)C3=C(C(=CC(=C3C2=O)O)C4=CC(=C5C(=C4OC)C(=O)C6=C(C5=O)CC(C(C6O)(C)O)O)O)OC |
| 70 | **Xestoquinol sulfate** | lipid extract of the marine sponge Petrosia alfiani |  | CMNPD5519 | 23427322 | CC12CCCC3=COC(=C31)C(=O)C4=CC5=C(C=CC(=C5C=C24)OS(=O)(=O)O)O |
| 71 | **deoxybostrycin** | mangrove endophytic fungus Nigrospora sp. No. 1403 |  | CMNPD22113 | 193579 | CC1(CC2=C(CC1O)C(=C3C(=O)C=C(C(=O)C3=C2O)OC)O)O |
| 72 | **11-Dehydrosinulariolide** | soft coral Sinularia leptoclados |  | CMNPD1325 | 137628341 | CC1=CCCC2(C(O2)CC3CCC(C(=O)CC1)(OC(=O)C3=C)C)C |
| 73 | **β-sitosterol** | marine green alga Tydemania expeditionis |  | CMNPD25328 | 222284 | CCC(CCC(C)C1CCC2C1(CCC3C2CC=C4C3(CCC(C4)O)C)C)C(C)C |
| 74 | **Stellettin A** | South China Sea sponge, Geodia japonica. |  | CMNPD6975 | 5352083 | CC1=CC=C(OC1=O)C(=CC=CC(=C2C(=O)CC3C2(CCC4C3(CCC(=O)C4(C)C)C)C)C)C |
| 75 | Fuscocineroside C | cucumber Holothuria scabra |  | CMNPD16499 | 44559164 | CC1C(C(C(C(O1)OC2C(C(COC2OC3CCC4(C(C3(C)C)CCC5C4=CC(C67C5(CCC6C(OC7=O)(C)C8CCC(O8)(C)C)C)O)C)OS(=O)(=O)[O-])O)O)O)OC9C(C(C(C(O9)CO)O)OC1C(C(C(C(O1)CO)O)OC)O)O.[Na+] |
| 76 | **Hirsutanol A** | marine fungus Chondrostereum sp. in the coral Sarcophyton tortuosum | Sesquiterpene Compounds | CMNPD9408 | 642919 | CC1(CC2(C(=CC3=CC(=O)C(=C)C32C)C1O)O)C |
| 77 | **chondrosterin A** | marine fungus Chondrostereum sp., |  | CMNPD22085 | 56962379 | CC1(CC2C3(C(=C)C(=O)C=C3CC2(C1)O)C)C |
| 78 | **chondrosterin B** | ''''''''''''''''''''''''''''''''''''''''''''''''''''''''''''''''''' |  | CMNPD22086 | 139585750 | CC1C(=O)C=C2C1(C3(CC(C(=O)C3=C2)(C)C)O)C |
| 79 | **chondrosterin C** | '''''''''''''''''''''''''''''''''''''''''''''''''''''''''''''' |  | CMNPD22087 | 139586332 | CC1C(=O)CC2C1(C3=C(C2O)C(=O)C(C3)(C)C)C |
| 80 | **chondrosterin D** | ''''''''''''''''''''''''''''''''''''''''''''''''''''''''''' |  | CMNPD22088 | 139587605 | CC1C(=O)CC2C1(C3=C(C2=O)C(=O)C(C3)(C)C)C |
| 81 | **chondrosterin E** | ''''''''''''''''''''''''''''''''''''''''''''''''''''''''''''''' |  | CMNPD22089 | 139583666 | CC1=CC(=O)C2(C1C3CC(CC3C2O)(C)C)C |
| 82 | **hirsutanol C** | marine fungus Chondrostereum sp., |  | CMNPD9410 | 10514702 | CC1C(=O)C=C2C1(C3(CC(C(C3=C2)O)(C)C)O)C |
| 83 | 24-Dehydroechinoside A | cucumber Holothuria scabra |  | CMNPD1718 | 101610326 | CC1C(C(C(C(O1)OC2C(C(COC2OC3CCC4(C(C3(C)C)CCC5C4=CC(C67C5(CCC6(C(OC7=O)(C)CCC=C(C)C)O)C)O)C)OS(=O)(=O)[O-])O)O)O)OC8C(C(C(C(O8)CO)O)OC9C(C(C(C(O9)CO)O)OC)O)O.[Na+] |
| 84 | **Laminarin** | Laminaria japonica Aresch (Laminariaceae) and Ecklonica kurome Okam |  | **NO RESULT** | 439306 | C(C1C(C(C(C(O1)O)O)OC2C(C(C(C(O2)CO)O)OC3C(C(C(C(O3)CO)O)O)O)O)O)O |
| 85 | **PBQ1** | marine algae Cymopolia barbata, |  | **NO RESULT** | 11024725 | CC1(CC(=O)C2=CC(=C(C=C2O1)Br)O)CCCC(C)(C)O |
| 86 | **PBQ2** | marine algae Cymopolia barbata, |  | **NO RESULT** | 71463496 | CC(=CC(=O)C1=CC(=C(C=C1O)Br)O)CCCC(C)(C)O |
| 87 | **SZ-685C(Haloroquinone)** | mangrove endophytic fungus No. 1403 |  | CMNPD19865 | 12301973 | CC1(CC2=C(C(C1O)O)C(=C3C(=O)C=C(C(=O)C3=C2O)OC)O)O |
| 88 | **altersolanol B** | Alternaria sp. ZJ-2008003, a fungus |  | CMNPD24949 | 161389 | CC1(CC2=C(CC1O)C(=O)C3=C(C2=O)C=C(C=C3O)OC)O |
| 89 | **altersolanol C** | Alternaria sp. ZJ-2008003, a fungus |  | CMNPD21998 | 171817 | CC1(C(CC2=C(C1O)C(=O)C3=C(C2=O)C(=CC(=C3)OC)O)O)O |
| 90 | **altersolanol L** | Alternaria sp. ZJ-2008003, a fungus |  | CMNPD24950 | 42639666 | CC1(C(CC2C(C1O)C(C3=C(C2=O)C(=CC(=C3)OC)O)O)O)O |
| 91 | **macrosporin** | ''''''''''''''''''''''''''''''''''''''''''''''''''''''''''''''''''''''' |  | CMNPD24948 | 159926 | CC1=CC2=C(C=C1O)C(=O)C3=C(C2=O)C=C(C=C3O)OC |
| 92 | **alterporriol C** | '''''''''''''''''''''''''''''''''''''''''''''''''''''''''''''''''''''''''''' |  | **NO RESULT** | 189544 | CC1=CC2=C(C(=C1O)C3=C(C=C4C(=C3O)C(=O)C5=C(C4=O)C(C(C(C5O)O)(C)O)O)OC)C(=O)C6=C(C2=O)C=C(C=C6O)OC |
| 93 | **fucosterol** | marine green alga Tydemania expeditionis |  | **NO RESULT** | 5281328 | CC=C(CCC(C)C1CCC2C1(CCC3C2CC=C4C3(CCC(C4)O)C)C)C(C)C |
| 94 | **saringosterol** | marine green alga Tydemania expeditionis |  | **NO RESULT** | 14161394 | CC(C)C(CCC(C)C1CCC2C1(CCC3C2CC=C4C3(CCC(C4)O)C)C)(C=C)O |
| 95 | **14-hydroxymethylxestoquinone** | lipid extract of the marine sponge Petrosia alfiani |  | CMNPD22480 | 71454859 | CC12CCCC3=COC(=C31)C(=O)C4=C2C=C5C(=C4)C(=O)C(=CC5=O)CO |
| 96 | **15-hydroxymethylxestoquinone** | lipid extract of the marine sponge Petrosia alfiani |  | CMNPD22481 | 71453127 | CC12CCCC3=COC(=C31)C(=O)C4=C2C=C5C(=C4)C(=O)C=C(C5=O)CO |
| 97 | **Xestoquinone** | lipid extract of the marine sponge Petrosia alfiani |  | CMNPD1956 | 122838 | CC12CCCC3=COC(=C31)C(=O)C4=C2C=C5C(=O)C=CC(=O)C5=C4 |
| 98 | **14, 15-dihydroxestoquinone** | lipid extract of the marine sponge Petrosia alfiani |  | NO RESULT | 71456630 | CC12CCCC3=COC(=C31)C(=O)C4=C2C=C5C(=O)CCC(=O)C5=C4 |
| 99 | **Adociaquinone A** | lipid extract of the marine sponge Petrosia alfiani |  | CMNPD3305 | 10364986 | CC12CCCC3=COC(=C31)C(=O)C4=C2C=C5C(=C4)C(=O)C6=C(C5=O)NCCS6(=O)=O |
| 100 | **Adociaquinone B** | lipid extract of the marine sponge Petrosia alfiani |  | CMNPD3306 | 11201041 | CC12CCCC3=COC(=C31)C(=O)C4=C2C=C5C(=C4)C(=O)C6=C(C5=O)S(=O)(=O)CCN6 |
| 101 | **cytarabine** | marine-derived pharmaceutical substances |  | **NO RESULT** | 6253 | C1=CN(C(=O)N=C1N)C2C(C(C(O2)CO)O)O |
| 102 | eribulin mesylate | marine-derived pharmaceutical substances |  | **NO RESULT** | 17755248 | CC1CC2CCC3C(=C)CC(O3)CCC45CC6C(O4)C7C(O6)C(O5)C8C(O7)CCC(O8)CC(=O)CC9C(CC(C1=C)O2)OC(C9OC)CC(CN)O.CS(=O)(=O)O |
| 103 | **plinabulin** |  |  | **NO RESULT** | 9949641 | CC(C)(C)C1=C(N=CN1)C=C2C(=O)NC(=CC3=CC=CC=C3)C(=O)N2 |
| 104 | plitidepsin |  |  | **NO RESULT** | 9812534 | CCC(C)C1C(CC(=O)OC(C(=O)C(C(=O)NC(C(=O)N2CCCC2C(=O)N(C(C(=O)OC(C(C(=O)N1)NC(=O)C(CC(C)C)N(C)C(=O)C3CCCN3C(=O)C(=O)C)C)CC4=CC=C(C=C4)OC)C)CC(C)C)C)C(C)C)O |
| 105 | lurbinectedin |  |  | **NO RESULT** | 57327016 | CC1=CC2=C(C3C4C5C6=C(C(=C7C(=C6C(N4C(C(C2)N3C)O)COC(=O)C8(CS5)C9=C(CCN8)C2=C(N9)C=CC(=C2)OC)OCO7)C)OC(=O)C)C(=C1OC)O |
| 106 | **marizomib** |  |  | **NO RESULT** | 11347535 | CC12C(C(=O)NC1(C(=O)O2)C(C3CCCC=C3)O)CCC |
| 107 | bryostatin |  |  | CMNPD1541 | 5280757 | CCCC=CC=CC(=O)OC1C(=CC(=O)OC)CC2CC(OC(=O)CC(CC3CC(C(C(O3)(CC4CC(=CC(=O)OC)CC(O4)C=CC(C1(O2)O)(C)C)O)(C)C)OC(=O)C)O)C(C)O |
| 108 | **cryptosphaerolide** | ascomycete strain CNL-523 (Cryptosphaeria sp.) | ester-substituted sesquiterpenoid | CMNPD19787 | 46831414 | CCC(C)CC(C)CC(CO)(C(=O)OC1CCC(C2(C13C(O3)C4(C(C2)C(=C)CO4)O)C)C)O |
| 109 | **manzamine A** | marine sponges | alkaloid | CMNPD3270 | 6509753 | C1CCN2CCC3C(=CC(CCC=CC1)(C4C3(C2)CC5N4CCCCC=C5)O)C6=NC=CC7=C6NC8=CC=CC=C78 |
| 110 | **Cholestane** | sea whip Leptogorgia sarmentosa |  | CMNPD2049 | 6857534 | CC(C)CCCC(C)C1CCC2C1(CCC3C2CCC4C3(CCCC4)C)C |
| 111 | Malyngamide C | cyanobacterium Lynbya majuscula | chlorinated amide derivative | CMNPD2192 | 20847479 | CCCCCCCC(CC=CCCC(=O)NCC(=CCl)C12C(O1)C(CCC2=O)O)OC |
| 112 | **hymenialdisine** | Marine sponges | bromopyrrole alkaloids | CMNPD4316 | 135413546 | C1CNC(=O)C2=C(C1=C3C(=O)NC(=N3)N)C=C(N2)Br |
| 113 | **sceptrin** | Marine sponges | bromopyrrole alkaloids | CMNPD982 | 157394 | C1=C(NC=C1Br)C(=O)NCC2C(C(C2C3=CN=C(N3)N)C4=CN=C(N4)N)CNC(=O)C5=CC(=CN5)Br |
| 114 | **Nakamuric acid** | marine sponges |  | CMNPD10443 | 9987214 | C1=C(NC=C1Br)C(=O)NCC2C(C(C2C3=CN=C(N3)N)C(=O)O)CNC(=O)C4=CC(=CN4)Br.C(=O)(C(F)(F)F)O |
| 115 | **debromosceptrin** | marine sponges |  | CMNPD4881 | 44584043 | C1=CNC(=C1)C(=O)NCC2C(C(C2C3=CN=C(N3)N)C4=CN=C(N4)N)CNC(=O)C5=CC=CN5.Cl.Cl |
| 116 | **hyrtiocarboline** | marine sponge |  | CMNPD20160 | 46184324 | C1=CC2=C(C=C1O)C3=CC(=NC(=C3N2)C(=O)C4=CN=CN4)C(=O)O |
| 117 | **norharman** | marine sponge |  | **NO RESULT** | 64961 | C1=CC=C2C(=C1)C3=C(N2)C=NC=C3 |
| 118 | **prostaglandin A1** |  | cyclopentenones | **NO RESULT** | 5281912 | CCCCCC(C=CC1C=CC(=O)C1CCCCCCC(=O)O)O |
| 119 | **prostaglandin A2** |  |  | CMNPD2951 | 5280880 | CCCCCC(C=CC1C=CC(=O)C1CC=CCCCC(=O)O)O |
| 120 | **prostaglandin J2** |  |  | **NO RESULT** | 5280884 | CCCCCC(C=CC1C(C=CC1=O)CC=CCCCC(=O)O)O |
| 121 | **didemnenone** | ascidian Lissoclinum sp., and from the marine-derived fungus Trichoderma sp. |  | CMNPD3534 | 101541749 | CC=CC(=C1C(=O)C=CC1(CO)O)CO |
| 122 | **Trichoderone** | ascidian Lissoclinum sp., and from the marine-derived fungus Trichoderma sp |  | CMNPD19842 | 44820524 | CCC1=CC(=O)C(C1O)O |
| 123 | **heteronemin** | sponges Heteronema erecta and Hytios sp. |  | CMNPD2007 | 21589810 | CC(=O)OC1CC2C3(CCC4C(CCCC4(C3CC(C2(C5C1=COC5OC(=O)C)C)O)C)(C)C)C |
| 124 | **Latrunculin A** | Red Sea sponge Negombata magnifica |  | CMNPD944 | 445420 | CC1CCC2CC(CC(O2)(C3CSC(=O)N3)O)OC(=O)C=C(CCC=CC=C1)C |
| 125 | **Latrunculin B** | Red Sea sponge Negombata magnifica |  | CMNPD945 | 6436219 | CC1CCC2CC(CC(O2)(C3CSC(=O)N3)O)OC(=O)C=C(CCC=C1)C |
| 126 | **Dermacozine A** | marine actinomycetes |  | CMNPD19656 | 46223672 | CN1C2=CC=CC(=C2NC3=CC=CC(=C31)C(=O)N)C(=O)N |

**Table S2. Properties 0f non-toxic drug like CMNPD compounds**

| **Compounds** | **Properties** | | | | |  |  |  |  |  |  |  |
| --- | --- | --- | --- | --- | --- | --- | --- | --- | --- | --- | --- | --- |
| **Factors** | **Molecular Weight** | **ALOGP** | **H-Bond Aceptor** | **H-Bond Donor** | **Rotatable Bonds** | **Drug likeness** | **Mutageneic** | **Tumorigeneic** | **Reproductivity effect** | **Irritant** | **CMNPS ID** | **PUBMED ID** |
| 10-acetylirciformonin B | 418.53 | 4.66 | 6 | 1 | 11 | -4.6296 | none | none | none | none | CMNPD21380 | 54669795 |
| 11-Dehydrosinulariolide | 332.44 | 3.89 | 4 | 0 | 0 | -4.6296 | none | none | none | none | CMNPD1325 | 137628341 |
| 14-hydroxymethylxestoquinone | 348.35 | 2.76 | 5 | 1 | 1 | -2.3233 | none | none | none | none | CMNPD22480 | 71454859 |
| 15-acetylirciformonin B | 418.53 | 4.66 | 6 | 1 | 11 | -1.1917 | none | none | none | none | CMNPD21379 | 54669794 |
| 15-hydroxymethylxestoquinone | 348.35 | 2.76 | 5 | 1 | 1 | -5.4008 | none | none | none | none | CMNPD22481 | 71453127 |
| Acetylapoaranotin | 488.54 | 1.78 | 9 | 0 | 2 | 4.8377 | low | low | none | none | NO RESULT | 9826799 |
| Adociaquinone A | 423.45 | 2.07 | 7 | 1 | 0 | -2.6272 | none | none | none | none | CMNPD3305 | 10364986 |
| Adociaquinone B | 423.45 | 2.07 | 7 | 1 | 0 | -2.6272 | none | none | none | none | CMNPD3306 | 11201041 |
| Agelasine | 458.09 | 2.37 | 4 | 1 | 5 | -2.077 | none | none | none | none | CMNPD2011 | 16667745 |
| altersolanol L | 324.33 | -0.26 | 7 | 5 | 1 | 2.9126 | none | none | none | none | CMNPD24950 | 42639666 |
| Aspergillide A | 254.33 | 1.96 | 4 | 1 | 0 | -8.1566 | none | none | none | none | CMNPD18899 | 70678632 |
| Bostrycin | 336.3 | -0.29 | 8 | 5 | 1 | 3.7308 | low | none | none | none | CMNPD17636 | 10042612 |
| chondrosterin B | 246.31 | 1.81 | 3 | 1 | 0 | -0.054338 | none | none | none | none | CMNPD22086 | 139585750 |
| chondrosterin C | 248.32 | 1.89 | 3 | 1 | 0 | -1.612 | none | none | none | none | CMNPD22087 | 139586332 |
| chondrosterin D | 246.31 | 2.1 | 3 | 0 | 0 | -1.7853 | none | none | none | none | CMNPD22088 | 139587605 |
| chondrosterin E | 234.34 | 2.56 | 2 | 1 | 0 | -1.6468 | none | none | none | none | CMNPD22089 | 139583666 |
| chondrosterins A | 232.32 | 2.63 | 2 | 1 | 0 | -3.5226 | none | none | none | none | CMNPD22085 | 56962379 |
| **cryptosphaerolide** | 466.62 | 2.95 | 7 | 3 | 8 | 9.2262 | none | none | none | none | CMNPD19787 | 46831414 |
| cytarabine | 243.22 | -2.56 | 8 | 4 | 2 | -4.6735 | none | none | none | none | **NO RESULT** | 6253 |
| deoxybostrycin | 320.3 | 0.22 | 7 | 4 | 1 | 3.9741 | low | none | none | none | CMNPD22113 | 193579 |
| **Dermacozine A** | 282.3 | 1.71 | 4 | 3 | 2 | 1.4825 | none | none | none | none | CMNPD19656 | 46223672 |
| **didemnenone** | 210.23 | -0.29 | 4 | 3 | 3 | -0.9815 | none | none | none | none | CMNPD3534 | 101541749 |
| Hippolide A | 415.57 | 4.88 | 4 | 2 | 10 | 2.1313 | none | none | none | none | CMNPD21383 | 53262772 |
| Hirsutanol A | 246.31 | 1.52 | 3 | 2 | 0 | -2.7808 | none | none | none | none | CMNPD9408 | 642919 |
| hirsutanol C | 248.32 | 1.6 | 3 | 2 | 0 | 1.5281 | none | none | none | none | CMNPD9410 | 10514702 |
| **hymenialdisine** | 324.14 | 0.07 | 4 | 4 | 0 | 0.3913 | none | none | none | none | CMNPD4316 | 135413546 |
| **hyrtiocarboline** | 322.28 | 2.07 | 5 | 4 | 3 | 1.6146 | low | none | none | none | CMNPD20160 | 46184324 |
| Hyrtioreticulins A | 312.33 | 1.48 | 4 | 5 | 3 | -0.33629 | none | none | none | none | **NO RESULT** | 70694527 |
| Hyrtioreticulins B | 312.33 | 1.48 | 4 | 5 | 3 | -0.33629 | none | none | none | none | **NO RESULT** | 70684035 |
| Irciformonin B | 376.49 | 4.09 | 5 | 2 | 10 | -5.317 | none | none | none | none | CMNPD21381 | 54669792 |
| Isogranulatimide | 276.25 | 2.02 | 5 | 2 | 0 | 4.4469 | none | none | none | none | CMNPD10017 | 135418335 |
| **Latrunculin A** | 421.56 | 3.86 | 6 | 2 | 1 | -9.882 | none | none | none | none | CMNPD944 | 445420 |
| **Latrunculin B** | 395.52 | 3.3 | 6 | 2 | 1 | -10.08 | none | none | none | none | CMNPD945 | 6436219 |
| Mycoepoxydiene | 290.32 | 1.55 | 5 | 0 | 2 | -1.4808 | none | none | none | none | **NO RESULT** | 11300750 |
| PBQ1 | 357.24 | 3.82 | 4 | 2 | 4 | -4.7311 | none | none | none | none | **NO RESULT** | 11024725 |
| PBQ2 | 357.24 | 3.93 | 4 | 3 | 6 | -6.8881 | none | none | none | none | **NO RESULT** | 71463496 |
| plinabulin | 336.4 | 0.74 | 3 | 3 | 2 | -1.1515 | none | none | none | none | **NO RESULT** | 9949641 |
| **prostaglandin A1** | 336.47 | 4.28 | 3 | 2 | 13 | -16.547 | none | none | none | none | **NO RESULT** | 5281912 |
| **prostaglandin A2** | 334.46 | 4.06 | 3 | 2 | 12 | 14.262 | none | none | none | none | CMNPD2951 | 5280880 |
| **prostaglandin J2** | 334.46 | 4.06 | 3 | 2 | 12 | -14.421 | none | none | none | none | **NO RESULT** | 5280884 |
| Protuboxepin A | 377.44 | 3.16 | 5 | 1 | 4 | 5.9163 | none | none | none | none | **NO RESULT** | 53355697 |
| Smenospongine | 343.47 | 3.98 | 4 | 2 | 2 | -9.2608 | none | none | none | none | CMNPD3299 | 3081931 |
| SZ-685C(Haloroquinone) | 336.3 | -0.29 | 8 | 5 | 1 | 3.7308 | low | none | none | none | CMNPD19865 | 12301973 |
| tetrahydroaltersolanol B | 308.33 | 0.77 | 6 | 4 | 1 | 3.2188 | none | none | none | none | **NO RESULT** | 70683135 |
| **Trichoderone** | 142.15 | -0.37 | 3 | 2 | 1 | 2.0903 | none | none | none | none | CMNPD19842 | 44820524 |
| Xestoquinol sulfate | 400.41 | 3.51 | 6 | 2 | 2 | -1.7094 | none | none | none | none | CMNPD5519 | 23427322 |
| Xestoquinone | 318.33 | 3.4 | 4 | 0 | 0 | -1.3517 | none | none | none | none | CMNPD1956 | 122838 |
